# Investigation of functional near-infrared spectroscopy signal quality and development of the hemodynamic phase correlation signal

**DOI:** 10.1117/1.NPh.9.2.025001

**Published:** 2022-05-18

**Authors:** Uzair Hakim, Paola Pinti, Adam J. Noah, Xian Zhang, Paul Burgess, Antonia Hamilton, Joy Hirsch, Ilias Tachtsidis

**Affiliations:** a University College London, Department of Medical Physics and Biomedical Engineering, London, United Kingdom; b University of London, Birkbeck College, Centre for Brain and Cognitive Development, London, United Kingdom; c Yale University, Department of Neuroscience and Comparative Medicine, Yale School of Medicine, United States; d University College London, Institute of Cognitive Neuroscience, London, United Kingdom

**Keywords:** functional near-infrared spectroscopy, hemodynamic response, neuroscience

## Abstract

**Significance:**

There is a longstanding recommendation within the field of fNIRS to use oxygenated (${\mathrm{HbO}}_{2}$) and deoxygenated (HHb) hemoglobin when analyzing and interpreting results. Despite this, many fNIRS studies do focus on ${\mathrm{HbO}}_{2}$ only. Previous work has shown that ${\mathrm{HbO}}_{2}$ on its own is susceptible to systemic interference and results may mostly reflect that rather than functional activation. Studies using both ${\mathrm{HbO}}_{2}$ and HHb to draw their conclusions do so with varying methods and can lead to discrepancies between studies. The combination of ${\mathrm{HbO}}_{2}$ and HHb has been recommended as a method to utilize both signals in analysis.

**Aim:**

We present the development of the hemodynamic phase correlation (HPC) signal to combine ${\mathrm{HbO}}_{2}$ and HHb as recommended to utilize both signals in the analysis. We use synthetic and experimental data to evaluate how the HPC and current signals used for fNIRS analysis compare.

**Approach:**

About 18 synthetic datasets were formed using resting-state fNIRS data acquired from 16 channels over the frontal lobe. To simulate fNIRS data for a block-design task, we superimposed a synthetic task-related hemodynamic response to the resting state data. This data was used to develop an HPC-general linear model (GLM) framework. Experiments were conducted to investigate the performance of each signal at different SNR and to investigate the effect of false positives on the data. Performance was based on each signal’s mean T-value across channels. Experimental data recorded from 128 participants across 134 channels during a finger-tapping task were used to investigate the performance of multiple signals [${\mathrm{HbO}}_{2}$, HHb, HbT, HbD, correlation-based signal improvement (CBSI), and HPC] on real data. Signal performance was evaluated on its ability to localize activation to a specific region of interest.

**Results:**

Results from varying the SNR show that the HPC signal has the highest performance for high SNRs. The CBSI performed the best for medium-low SNR. The next analysis evaluated how false positives affect the signals. The analyses evaluating the effect of false positives showed that the HPC and CBSI signals reflect the effect of false positives on ${\mathrm{HbO}}_{2}$ and HHb. The analysis of real experimental data revealed that the HPC and HHb signals provide localization to the primary motor cortex with the highest accuracy.

**Conclusions:**

We developed a new hemodynamic signal (HPC) with the potential to overcome the current limitations of using ${\mathrm{HbO}}_{2}$ and HHb separately. Our results suggest that the HPC signal provides comparable accuracy to HHb to localize functional activation while at the same time being more robust against false positives.

## 1 Introduction

Functional near-infrared spectroscopy (fNIRS) is a noninvasive, optical neuroimaging modality that measures and maps the tissue concentrations changes of oxy- and deoxyhemoglobin ($\Delta {\mathrm{HbO}}_{2}$ and HHb), providing an estimate of the brain hemodynamic and oxygenation changes secondary to neuronal activation. Theoretically, brain activation in fNIRS is considered as a statistically significant increase in ${\mathrm{HbO}}_{2}$ and a decrease in HHb,^1^ based on the principle of neurovascular coupling, where brain activation is marked by an initial dip in HHb followed by a large increase of ${\mathrm{HbO}}_{2}$ in the blood to support the increased neurons’ metabolic demand. This increase in blood volume, which is composed of an increase in ${\mathrm{HbO}}_{2}$ and a smaller decrease in HHb, is called the hemodynamic response.

fNIRS offers some advantages over functional magnetic resonance imaging (fMRI), which by many is considered one of the gold standard neuroimaging methods. It has a higher temporal resolution, greater portability, increased tolerance to movements, and relatively lower cost. In particular, fNIRS allows more ecologically valid experimental tasks and wider application in populations such as infants and toddlers.

However, fNIRS presents some limitations. The emitted light must travel through multiple extra-cerebral layers (i.e., scalp, skull, and CSF) before reaching the brain and reflecting back onto the detector on the surface of the head. As a result, the light is attenuated through the various layers by the changes in the oxygen levels in the blood. This can be a significant limitation when fNIRS is used to investigate brain functions, which also leads to substantial changes in systemic physiology, such as partial pressure of ${\mathrm{CO}}_{2}$ in the blood, heart rate, blood pressure, or autonomic nervous system activity.^2^^–^^6^ These physiological responses can lead to changes in oxygenation independently of the neuronal activation, in both the cerebral and extracerebral layers. The end effect of this issue is the possibility of false positives and negatives arising when inferring functional brain activity from fNIRS signals.

This issue has been a longstanding problem in fNIRS analysis, brought to light by Obrig and Villringer.^7^ They specified that while an increase in ${\mathrm{HbO}}_{2}$ is part of the definition of neuronal activation, it may also reflect a change in blood pressure or an increase in skin blood volume. This was reinforced by Kirlilna et al.^8^ with quantitative results showing that the ${\mathrm{HbO}}_{2}$ signal is much more strongly affected by the systemic physiology. In review paper, Scholkmann et al.^9^ discussed this in more detail, and separated the fNIRS signal into six components: cerebral and extracerebral each with evoked and nonevoked neuronal and systemic components. Based on the analysis from Tachtsidis and Scholkmann,^2^ HHb seems to be more robust to systemic interferences than ${\mathrm{HbO}}_{2}$, and hence arguably more robust against false positives and negatives; however, HHb is not completely robust against false positives and negatives, therefore using it does not solve the issue of false positives and negatives. The reduced sensitivity to systemic interference is likely due to its smaller amplitude changes compared to ${\mathrm{HbO}}_{2}$, which in turn also means less statistical power. On the contrary, ${\mathrm{HbO}}_{2}$ is a high contrast signal, with larger task-related changes, but it seems to be more sensitive to physiological changes and thus more prone to false positives/negatives. Obrig and Villringer^7^ provided one possible way to account for this problem, which is to use the ${\mathrm{HbO}}_{2}$ signal in conjunction with HHb when inferring functional brain activity with fNIRS. This was reiterated again by Tachtsidis and Scholkmann again, 13 years later,^2^ where the authors state that it is of immediate importance for studies to report results from both ${\mathrm{HbO}}_{2}$ and HHb to allow better physiological interpretation of results. Most recently, the fNIRS best practices paper^10^ again recommended that both ${\mathrm{HbO}}_{2}$ and HHb need to be included when reporting and visualizing statistical results.

**Fig. 1:**
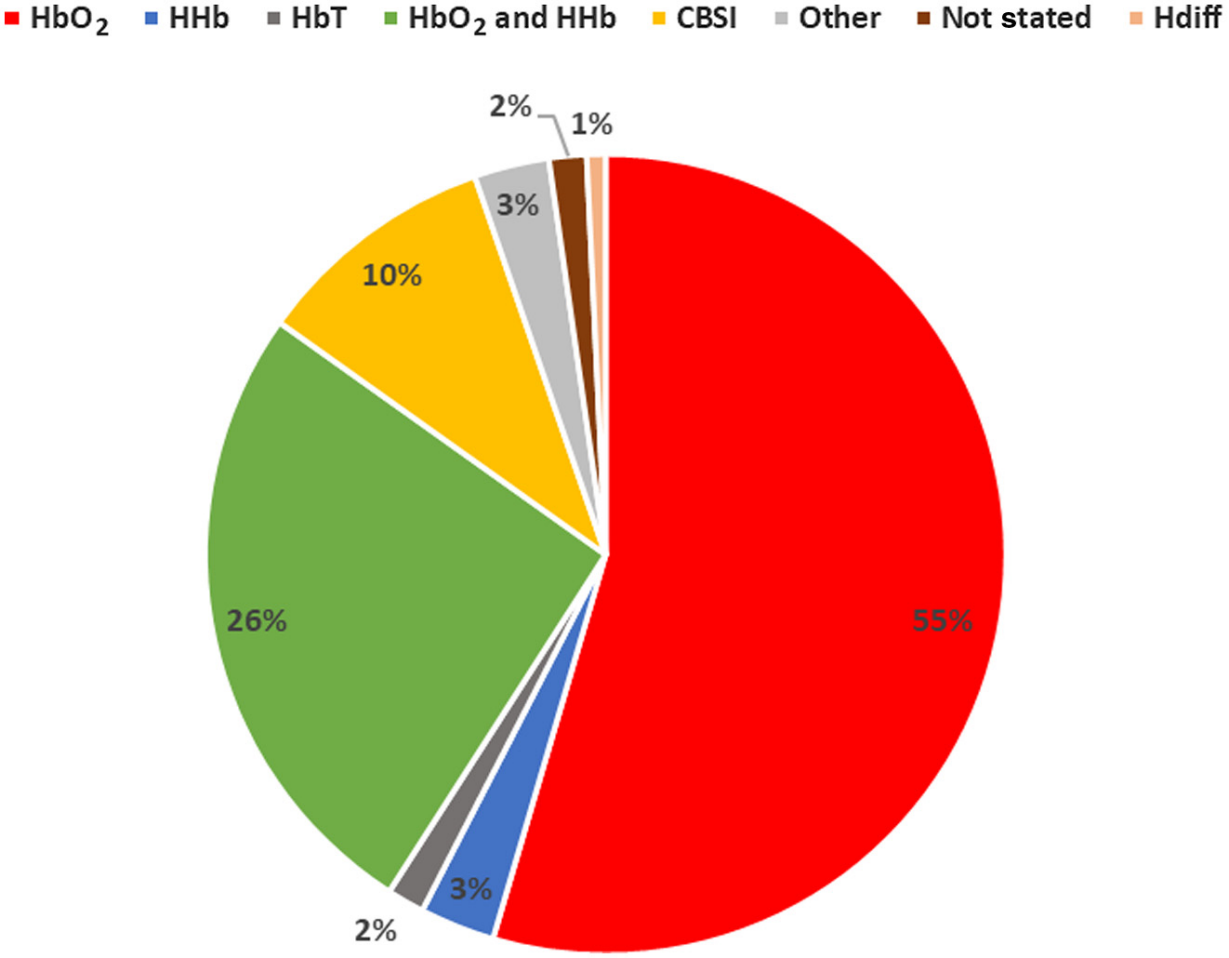
Percentage of studies using each signal. Peer-reviewed studies from 2017 to 2020 in the field of cognitive neuroscience using fNIRS were reviewed. Resting-state papers were excluded.

To investigate which fNIRS signals researchers used most often for inferring brain activation in the field of cognitive neuroscience, we looked at peer-reviewed studies published in the field from 2017 to 2020. A search was carried out on the ScienceDirect database using the keywords “fNIRS” and “cognitive neuroscience” (repeated with the abbreviation fNIRS) between 2017 and 2020, although the search parameters capture papers, they are not exhaustive of the field, as such this was not a systematic review. About 132 papers were reviewed once papers examining resting-state connectivity were excluded. After inspecting the methods and results section of each paper for which fNIRS-derived signal was used to assess the presence of significant task-related brain activity, we found that 55% of papers only used ${\mathrm{HbO}}_{2}$, and 3% of papers used HHb, and 26% of papers used both ${\mathrm{HbO}}_{2}$ and HHb. Figure 1 displays the percentages of each signal used and a full table can be found in Table S4 in the Supplemental Material.

The majority of the studies draw their conclusions based on an analysis of ${\mathrm{HbO}}_{2}$ only. This is usually because neuronal activation leads to a larger increase in ${\mathrm{HbO}}_{2}$, since neurons require more oxygen to cope with the energetic requirements, and a smaller decrease in HHb, giving ${\mathrm{HbO}}_{2}$ changes higher contrast and hence superior sensitivity in the evaluation of activity from baseline to task periods.^11^^–^^13^ In turn, this makes it easier to get statistically significant results using ${\mathrm{HbO}}_{2}$ and so studies are more likely to base their analysis on this signal only.

In contrast with this, only four studies used the HHb signal alone. Three of these studies provided justifications for their choice, such as the HHb ability to maximize spatial specificity,^14^ and its robustness to artifacts caused by extra-cerebral perfusion.^15^^,^^16^ These benefits of HHb have been quantified by studies investigating the physiological components of HHb and ${\mathrm{HbO}}_{2}$,^3^^,^^8^^,^^17^ while the basis of BOLD fMRI has been well established^18^ and its similarity to HHb has been shown Refs. 19–21. However, HHb typically has a lower magnitude and so there is less statistical power and larger sample sizes might be needed to observe a significant effect.

Promisingly, 34 studies^22^^–^^55^ did use both signals in some way; however, a lack of a common statistical framework to analyze both signals simultaneously has led to variation in how these signals were used. For example,^29^ considered activation as an “increase in ${\mathrm{HbO}}_{2}$ and/or a decrease in HHb.” In this case, both signals were analyzed statistically but interpretation based on only one signal. The authors found no significant activation in the HHb data and neuroscientific interpretations were based only on ${\mathrm{HbO}}_{2}$. In comparison (Wriessnegger, et al, 2017), conducted statistical analysis on the maxima of the concentration changes of ${\mathrm{HbO}}_{2}$ and the minima of HHb, after averaging over each trial per task.

Similar to de Klerk et al. (2018),^29^ Jackson et al. (2019)^34^ conducted four separate ANOVAs for two experimental conditions and ${\mathrm{HbO}}_{2}$ and HHb and presented results such that an increase in ${\mathrm{HbO}}_{2}$ or a decrease in HHb is used to determine activation. In this case, the authors analyzed both signals, but the interpretation was based on the signals separately. The lack of a framework to analyze and interpret both ${\mathrm{HbO}}_{2}$ and HHb has caused variability in methods and interpretations between studies.

One possible method of overcoming the problem of no framework to analyze both signals is to use one signal that has been derived using ${\mathrm{HbO}}_{2}$ and HHb. There are currently three such signals in the fNIRS field. Total hemoglobin (${\mathrm{Hb}}_{\mathrm{Tot}}={\mathrm{HbO}}_{2}+\mathrm{HHb}$), which can be used as an indication of blood volume changes. ${\mathrm{Hb}}_{\text{Total}}$ is expected to increase during neuronal activity with a smaller amplitude than ${\mathrm{HbO}}_{2}$. From the reviewed studies only two studies used ${\mathrm{Hb}}_{\text{Total}}$.^56^ justified their use of the signal as it incorporates the characteristics of neurovascular coupling from ${\mathrm{HbO}}_{2}$ and HHb, while^57^ did not provide any reasoning for its use. Hemoglobin difference (${\mathrm{Hb}}_{\mathrm{Diff}}={\mathrm{HbO}}_{2}-\mathrm{HHb}$) allows a measurement of oxygenation and identification of the mismatch between ${\mathrm{HbO}}_{2}$ and HHb. During neuronal activation, the signal should increase with an amplitude higher than ${\mathrm{HbO}}_{2}$, since HHb should be negative. Only one study used this signal.^58^ The authors justified its use using the same reasoning as Ref. 56 but did not provide specific reasoning for using ${\mathrm{Hb}}_{\mathrm{Diff}}$ rather than ${\mathrm{Hb}}_{\text{Total}}$. Although these signals do indeed combine information from ${\mathrm{HbO}}_{2}$ and HHb, systemic interference occurring in either ${\mathrm{HbO}}_{2}$ or HHb will still affect them. A typical marker of systemic interference is HHb increasing at the same time as ${\mathrm{HbO}}_{2}$. In this case, the ${\mathrm{Hb}}_{\text{Total}}$ will increase with an amplitude larger than ${\mathrm{HbO}}_{2}$. In turn, this will lead to false-positive activations being interpreted. The ${\mathrm{Hb}}_{\mathrm{Diff}}$ signal will show an amplitude lower than ${\mathrm{HbO}}_{2}$ in this case, causing false negatives since the amplitudes will be lower than expected.

Finally, the correlation-based signal improvement (CBSI) signal^59^ is a good example of a possible way to combine the ${\mathrm{HbO}}_{2}$ and HHb signals for the identification of brain activity, while trying to account for the brain physiological response to activation. The signal scales the correlation of ${\mathrm{HbO}}_{2}$ and HHb such that it enforces the anticorrelation and reduces any positive correlations. To do this it incorporates a scaling factor, which is the ratio of the standard deviations of ${\mathrm{HbO}}_{2}$ and HHb. In this paper, the authors show using experimental data that removes motion spikes, improves the CNR, and provides better spatial specificity, enforcing the idea that using the relationship of ${\mathrm{HbO}}_{2}$ and HHb to form a single signal can provide better localization of activity. Thirteen studies utilized the signal.^60^^–^^72^ The main justification was its ability to reduce motion and systemic artifacts; however Ref. 61 also specified its assumption of cortical activation being reflected by simultaneous increases and decreases in ${\mathrm{HbO}}_{2}$ and HHb. The key difference between the CBSI signal and ${\mathrm{Hb}}_{\mathrm{Diff}}$ and ${\mathrm{Hb}}_{\text{Total}}$ is the CBSI is formed based on an underlying characteristic of the hemodynamic response function (HRF) that is the anticorrelation between ${\mathrm{HbO}}_{2}$ and HHb.

As highlighted by the literature review, the majority of papers utilize ${\mathrm{HbO}}_{2}$ when analyzing fNIRS data, despite calls for the analysis of both ${\mathrm{HbO}}_{2}$ and HHb dating back to 2003. Nonetheless, there is heterogeneity in which fNIRS-derived signal is used to infer functional activation. In the studies using both signals, there is no standard framework for analyzing both signals together. In this paper, we aim to explore these issues. We provide initial investigations on which fNIRS-derived signal can predict brain activity most accurately. Additionally, we introduce and develop a novel signal that encodes phase information from ${\mathrm{HbO}}_{2}$ and HHb, named the hemodynamic phase correlation (HPC) signal. To achieve these goals, we compare the performance of the most used fNIRS-derived signals, namely the ${\mathrm{HbO}}_{2}$, HHb, CBSI, ${\mathrm{Hb}}_{\text{Total}}$, and ${\mathrm{Hb}}_{\mathrm{Diff}}$, alongside the newly proposed HPC signal.

Based on the recommendation of Refs. 2, 7, 9, and 10, the HPC signal utilizes information coming from ${\mathrm{HbO}}_{2}$ and HHb simultaneously. The HPC signal quantifies the phase relationship between ${\mathrm{HbO}}_{2}$ and HHb at each timepoint of the fNIRS recording. Specifically, the HPC uses the fundamental antiphase relationship between ${\mathrm{HbO}}_{2}$ and HHb to identify functional brain activity.

In this paper, we first present the development of the HPC signal. We then test the accuracy of ${\mathrm{HbO}}_{2}$, HHb, ${\mathrm{Hb}}_{\text{Total}}$, ${\mathrm{Hb}}_{\mathrm{Diff}}$, CBSI, and the HPC in identifying functional brain activity using a synthetic dataset of 18 subjects with 16 channels and experimental fNIRS data acquired from 128 adult participants during a finger-thumb tapping task using 134 channels.

## 2 Method

### 2.1 fNIRS Synthetic Data Generation

**Fig. 2:**
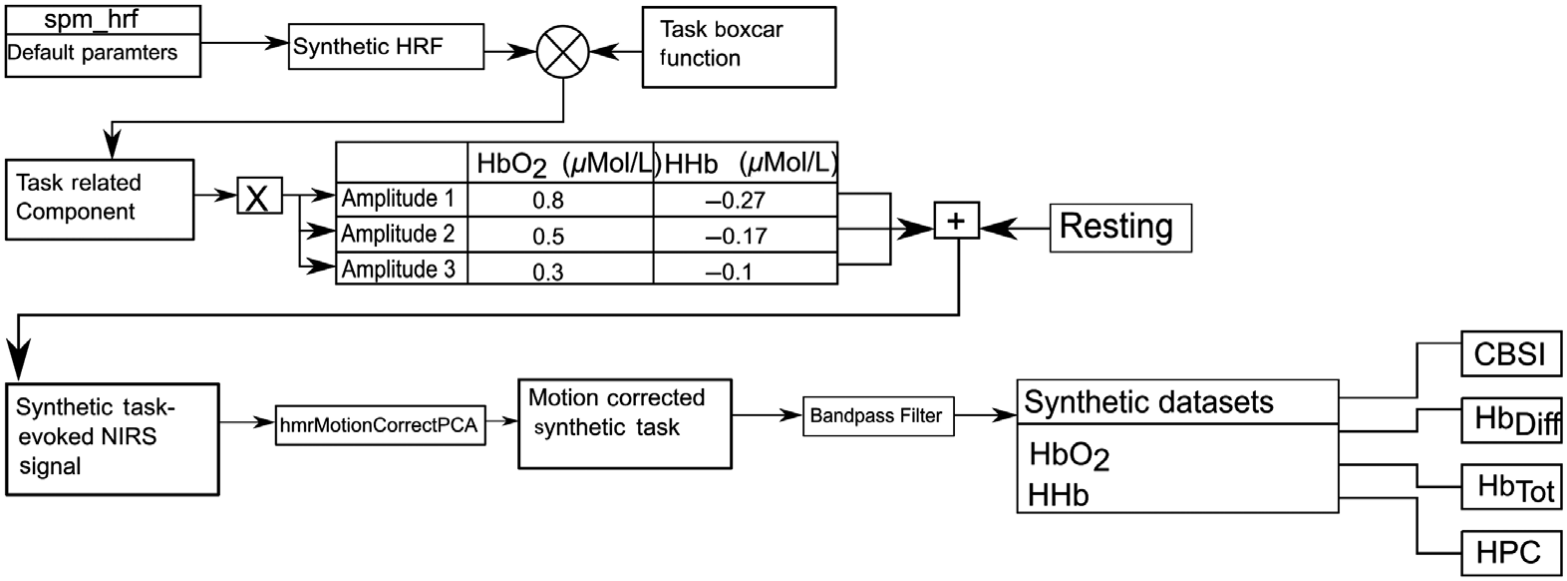
Synthetic data formation. Resting-state fNIRS data were obtained from the Hitachi WOT system using 16 channels at 5-Hz sampling frequency.

The generation of the synthetic data is shown in Fig. 2. The function “spm_hrf” part of the spm12 toolbox was used to generate a synthetic HRF with the default parameters. The function uses two gamma functions to form the HRF; a positive one that models the response and has a peak at 6 s after onset, and a negative one to model the undershoot, which has a minimum at 16 s. In particular, this is the canonical waveform reflecting the ${\mathrm{HbO}}_{2}$ response to a single functional event. The HHb signal is typically negative and with a smaller amplitude than the ${\mathrm{HbO}}_{2}$.^73^ The HHb signal, therefore, was formed by multiplying the canonical HRF amplitude (i.e., the ${\mathrm{HbO}}_{2}$ response) by $-\frac{1}{3}$ assuming HHb to have an amplitude 3 times smaller than ${\mathrm{HbO}}_{2}$. This procedure was repeated with three different ${\mathrm{HbO}}_{2}$ and HHb amplitudes [i.e., strong ($\mathrm{RMS}=4.8\times 10^{-7}$), mild ($\mathrm{RMS}=2.9\times 10^{-7}$), small ($\mathrm{RMS}=1.7\times 10^{-7}$) responses) to vary the SNR. To create the task-related component, a boxcar with a 40 s active condition and a 40 s rest condition was created to model a block-design task, and then convolved with the HRF. The task-related component was then added to resting state fNIRS data that approximates systemic noise. Actual systemic noise in an experiment may be highly correlated with task-block onsets and the method used here may not represent the task-induced noise but serves as an adequate approximation to it.

Resting-state fNIRS data were collected using the Hitachi wearable optical tomography (WOT, Hitachi High-Technologies Corporation, Japan) system at a sampling frequency of 5 Hz. Light is emitted via six light-sources at 705 and 830 nm. Light sources and detectors were spaced 3-cm apart, with one pairing constituting a single channel. Data were collected from 18 subjects with a 16-channels configuration across the frontal lobe. To ensure uniformity in channel positioning across participants, the 10/20 system was used, with channel 8 placed at the FpZ point and channels 8 and 9 aligned to the Nasion–Inion line. Data were collected for 10 min, while participants were sitting in a chair with their eyes closed. The channel configuration is shown in Fig. S11 in the Supplemental Material.

The raw fNIRS data were first converted to optical densities and then to concentration changes using the modified Beer–Lambert law.^74^ This data was then added to the task-related HRF and preprocessing was carried out on the output. Motion correction was applied using the PCA method from Ref. 75 with the default parameters, and a fourth-order Butterworth band-pass filter with frequency cut-offs at 0.01 and 0.2 Hz, these parameters were determined to be adequate for attenuating systemic interference by the filter analysis in Ref. 76. The filtered ${\mathrm{HbO}}_{2}$ and HHb signals were used to form the ${\mathrm{Hb}}_{\mathrm{Diff}}$, ${\mathrm{Hb}}_{\mathrm{Tot}}$, CBSI,^59^ and HPC signals. The peak amplitudes of each of the resulting signals for each amplitude variation are shown in Table 1. Following the process outlined in Fig. 2, the synthetic data consisted of 18 datasets with 16 channels each.

**Table 1:** Peak amplitudes for synthetic data conventional signals.

	Amplitude 1 (μ mol)	Amplitude 2 (μ mol)	Amplitude 3 (μ mol)
${\mathrm{HbO}}_{2}$	0.8	0.5	0.3
HHb	−0.27	−0.17	−0.1
CBSI	0.8	0.5	0.3
${\mathrm{Hb}}_{\mathrm{Diff}}$	1.07	0.67	0.4
${\mathrm{Hb}}_{\mathrm{Tot}}$	0.53	0.33	0.2

### 2.2 fNIRS Experimental Data Acquisition and Preprocessing

Experimental data were provided by the Brain Function Laboratory, Yale University.^77^ Hemodynamic signals were acquired using the LABNIRS System from Shimadzu Corporation (LABNIRS, Shimadzu Corporation, Japan). Data from 128 participants were used with mean age 25±7.6 years of age, 50% were female and 93% were right-handed. Signals were acquired from 134 channels covering the entire head (Fig. S12 in the Supplemental Material). Channels were localized using a 3D digitizing system and projected into the MNI space. Anatomical locations are shown in Table S5 in the Supplemental Material. The system acquired data at a sampling frequency of 8.1 Hz. The sources emit light at three wavelengths of 780, 805, and 830 nm. Caps were selected based on participant head size, with larger heads having a source–detector separation of 3 cm and smaller heads at 2.75 cm, to have comparable channel locations across subjects. Both caps had identical optode configurations. Assessment was made visually on the day, if the cap was to lose or tight a different sized cap was used. The experimental protocol was a right-handed finger thumb tapping task, chosen as it is a well-established protocol and serves as a reliable fiducial marker for signal comparisons. Participants were positioned 70 cm from a screen displaying instructions. A block design was used with alternating finger-thumb tapping and rest periods of 15 s each, repeated 6 times, for a total duration of 3 min.

Task periods included visual presentation of the numbers 1, 2, 3, or 4 in random order shown in black font in the center of a white screen. Numbers indicated which right-hand finger to firmly press against the right thumb and were presented every 0.75 s for a total of 20 numbers per active epoch. Participants were asked to respond as quickly and accurately as possible. In the case of mistakes, participants were told to continue without correcting. Rest epochs consisted of viewing a black crosshair presented in the center of a white screen. During rest epochs, participants were instructed to remain stationary without moving their fingers, clear their mind, and focus their eyes on the center of the screen. Participants were also told, in general, to refrain from taking deep breaths, keep their body still, and refrain from fidgeting and touching their face. The data were exported from the system as raw voltages and processed using functions from the Homer2 toolbox.^78^ The raw voltages were converted to optical density using the hmrIntensity2OD function and then motion-corrected using hmrMotionCorrectWavelet.^79^ The output was bandpass filtered using a Butterworth filter with order 5 and cut-offs at 0.01 and 0.2 Hz.^76^ Finally, hmrOD2Conc was used with differential pathlength factors of 6 for each wavelength. The final preprocessed ${\mathrm{HbO}}_{2}$ and HHb signals were used to compute the remaining signals, shown in Table 2.

**Table 2:** Equations used to form CBSI, ${\mathrm{Hb}}_{\mathrm{Diff}}$, and ${\mathrm{Hb}}_{\text{Total}}$.

Signal	Formula
CBSI	$\frac{1}{2}({\mathrm{HbO}}_{2}-\alpha \mathrm{HHb})$
${\mathrm{Hb}}_{\mathrm{Diff}}$	${\mathrm{HbO}}_{2}-\mathrm{HHb}$
${\mathrm{Hb}}_{\mathrm{Tot}}$	${\mathrm{HbO}}_{2}+\mathrm{HHb}$

### 2.3 Hemodynamic Phase Correlation (HPC) Computation

**Fig. 3:**
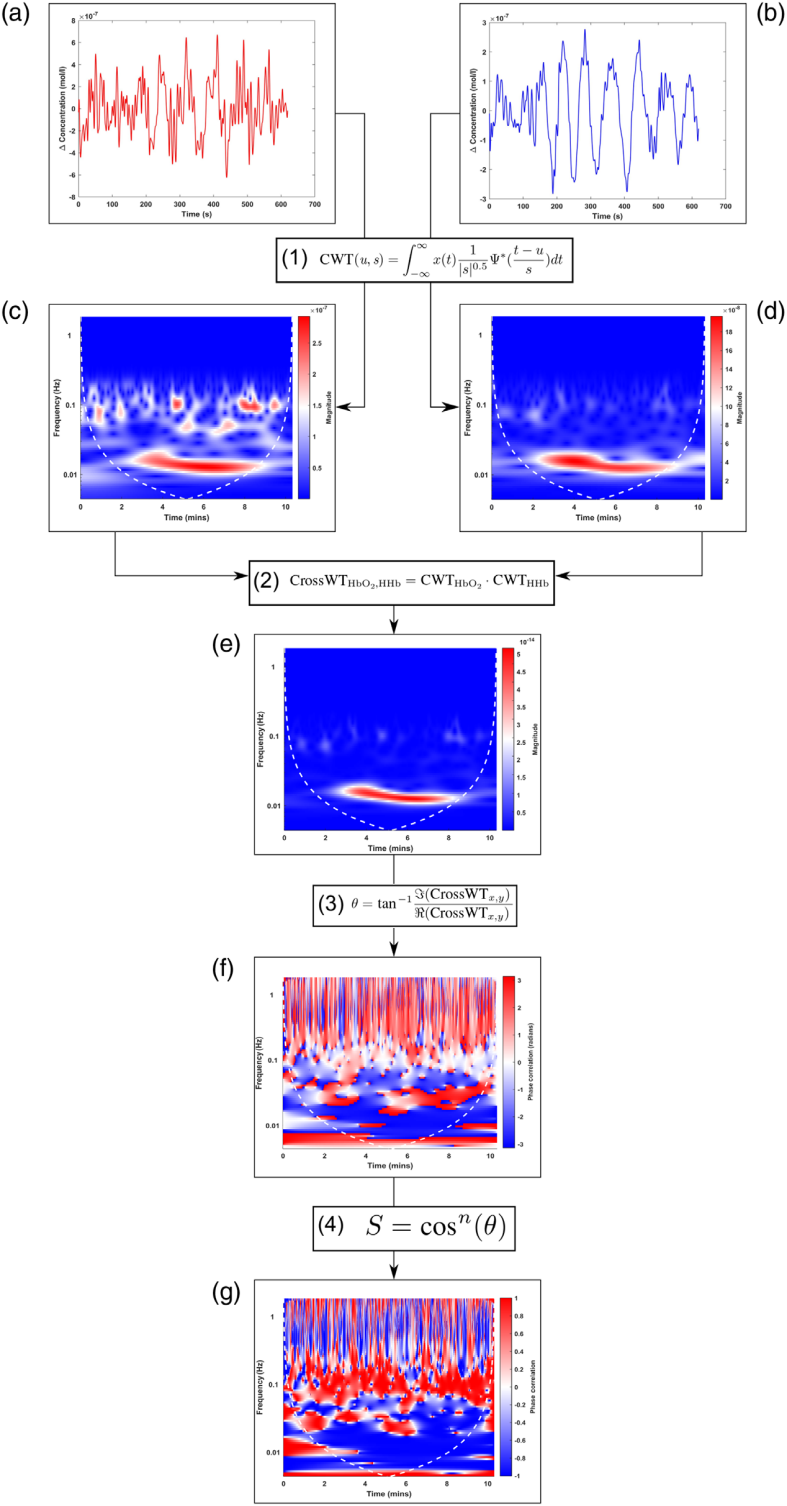
Generation of HPC signal from its component signals, (a) ${\mathrm{HbO}}_{2}$ and (b) HHb. A task frequency of 0.025 Hz was used. Denoted by the shaded areas in the time series and highlighted by a white box in the spectrograms. Continuous wavelet transform applied to both signals (c) and (d). Cross wavelet transform is computed (e) and the phase extracted is shown in (f), which is converted to a normalized value, the HPC, in (g).

The method outlined by Ref. 80 was used to form the HPC signal. The method transforms the time series data of ${\mathrm{HbO}}_{2}$ and HHb to the time-frequency domain and the phase correlation is then derived, the formation is shown in Fig. 3.

The first equation in Fig. 3 refers to the continuous wavelet transform where s is the scale, u is the displacement, Ψ is the mother wavelet used, and * refers to the complex conjugate. The mother wavelet used was the Morlet wavelet, in agreement with other fNIRS studies using wavelets.^3^^,^^4^^,^^81^^,^^82^ Once both CWTs are computed, they are used to compute the cross wavelet transform^83^ by multiplying the CWT of ${\mathrm{HbO}}_{2}$ with the complex conjugate of HHb. The output is a complex value with an amplitude A, and local phase θ, which is calculated using the third equation in Fig. 3. θ describes the phase correlation between the two signals at each frequency, for all time points in radians and ranges between −π and +π. One disadvantage of using solely the θ values is that the values of −π and +π symbolize the same thing, 180 deg out of phase: an anticorrelation between the two datasets. This can lead to a misevaluation of the data, especially if inspecting visually. For example, in Fig. 3(F), the row of data occurring at 0.015 Hz shows distinctive areas of +π and −π, in actual terms, these pertain to the same thing, an anticorrelated phase. By calculating the cosine of these values, we can use this information in a more useful way. The values now range from −1 to +1 (anticorrelated, to correlated) representing the information in a more interpretable way. The parameter n in Eq. (4) of Fig. 3 is an odd integer >0 and can be altered to sharpen the response of the HPC by reducing the smearing of an area where there is a strong positive or negative correlation. In this case, the value of n was left at 1 as at this value there was no smearing and the output provided responses that were expected. The output of this is shown in Fig. 3(g).

The y-axis of the spectrograms shows the scale parameter alongside the frequency. The scale refers to how the mother wavelet is scaled when fitting the signal. The scale can be converted to a “pseudofrequency” using the following equation:^84^

$$F_{a}=\frac{F_{c}}{a\cdot \Delta },$$ (1)

where “a” is the scale, $F_{a}$ is the pseudofrequency of a corresponding scale “a,” $F_{c}$ is the center frequency of the wavelet in Hz and Δ is the sampling period. As we are determining the frequency based on the scale, in the text only frequencies will be used to avoid over-complication; however, in the figures both scale and frequency are shown.

Since the method utilizes wavelet transforms, the issue of edge-effect artifacts is present. These occur when the scaled wavelet extends beyond the edges of the observation interval. These regions are delineated in all spectrograms by the dashed-white line, known as the cone-of-influence (COI). Regions within the COI are potentially affected by edge effects. However, as shown in Figs. 5 and 6, the task frequencies used for the simulated data minimally overlap with the COI. Additionally, the experimental data is a higher frequency than the simulated and since the COI is larger for lower frequencies (low frequency = high scale = extends past signal), the higher the frequency the less the signal exists within the COI. However, in the case where the task frequency has a large overlap within the COI, the user should state how much of the signal is overlapping within the COI.

#### 2.3.1 ${\mathrm{HbO}}_{2}$ decreasing and HHb increasing (reversal pattern)

A possible source of error using the HPC signal may arise when the ${\mathrm{HbO}}_{2}$ signal is decreasing and the HHb is increasing during functional activation. Since this is still antiphase, the HPC will detect it as activation, although it is not. This reversal hemodynamic pattern may be of physiological interest, but it is a confounding factor in the HPC-based detection of functional activation. To account for this, we developed an add-on function able to detect functional blocks where this reversed response pattern occurs. This method is based on the Hbdiff signal (${\mathrm{Hb}}_{\mathrm{Diff}}={\mathrm{HbO}}_{2}-\mathrm{HHb}$). During functional activation, where ${\mathrm{HbO}}_{2}$ is increasing (positive) and HHb is decreasing (negative), the ${\mathrm{Hb}}_{\mathrm{Diff}}$ signal should be positive; instead, in the case of ${\mathrm{HbO}}_{2}$ being negative and HHb positive (i.e., the reversed response pattern), ${\mathrm{Hb}}_{\mathrm{Diff}}$ is negative. Therefore, detecting the negative trend of ${\mathrm{Hb}}_{\mathrm{Diff}}$ can be used to determine where and when the reversal pattern occurs.

The add-on function proposed here first computes the ${\mathrm{Hb}}_{\mathrm{Diff}}$ signal within each task block and then computes the area of the regions bounded by the ${\mathrm{Hb}}_{\mathrm{Diff}}$ signal and the x axis. In this way, we quantify the ratio between the areas of the positive and negative regions of the ${\mathrm{Hb}}_{\mathrm{Diff}}$ signal during the functional stimulation period. If the ${\mathrm{Hb}}_{\mathrm{Diff}}$ signal during the stimuli block is dominated by the reversed response pattern, there should be much more negative area than positive, resulting in a ratio <0.5; once this is detected, the affected block can be automatically removed from further analyses or manually inspected and removed by the user. The 0.5 ratio threshold was empirically chosen by using our synthetic data while on purpose implementing hemodynamic reversal response patterns. This ratio was a good compromise in accurately distinguishing a true hemodynamic reversal response pattern versus post-stimulus hemodynamic undershoots, where ${\mathrm{HbO}}_{2}$ and HHb are reversed as part of the cerebral hemodynamic response to stimuli.

### 2.4 Data Analysis

Synthetic and experimental data were analyzed using the general linear model (GLM). This method was developed for the analysis of fMRI data by Ref. 85 and extended to fNIRS. It expresses the fNIRS signal as a linear combination of several variables, known as regressors, plus an error term. The GLM is the preferable method of analysis as it considers the entire time series of the data and allows the inclusion of multiple variables to explain the measured signal (see Ref. 1 for a full review and explanation of the principles of the GLM). For ${\mathrm{HbO}}_{2}$, HHb, CBSI, ${\mathrm{Hb}}_{\mathrm{Tot}}$, ${\mathrm{Hb}}_{\mathrm{Diff}}$, the design matrix included the task regressor (either synthetic 40-s task blocks or 15-s finger tapping blocks) created through the convolution of the HRF with the boxcar representing the task onsets. For the experimental data task onset times were acquired using triggers implemented with the fNIRS acquisition software. The ${\mathrm{HbO}}_{2}$ version of the HRF was used for all signals apart from HHb, where a negative HRF was used, and the HPC where a specific HPC-RF was used (see Sec. 2.4.1).

#### 2.4.1 HPC specific data analysis

Since the HPC exists in the time-frequency domain and does not resemble a typical fNIRS signal, a specific framework for its application to the GLM was developed using the synthetic data. The data was formed using synthetic data with a peak ${\mathrm{HbO}}_{2}$ amplitude of $0.8\times 10^{-6}\;\mathrm{Mol}$ and peak HHb amplitude of $-0.27\times 10^{-6}\;\mathrm{Mol}$, since it has the highest SNR and the framework derived from it will have the strictest parameters for use with the experimental data.

In the design matrix of standard fNIRS analysis (i.e., ${\mathrm{HbO}}_{2}/\mathrm{HHb}$), the HRF convolved with a task-related boxcar function is used, called here the standard HRF (sHRF). In the case of HPC analysis, this method would not be suitable since the signal is not directly comparable to the HRF, rather it is a representation of the relationship between ${\mathrm{HbO}}_{2}$ and HHb. Therefore, to use the GLM with the HPC signal, a representation of this relationship should be used in the design matrix (comparable to standard analysis), we denote this as the HPC-RF. The HPC-RF is equal to the HPC of the sHRF of ${\mathrm{HbO}}_{2}$ and HHb. Since the sHRF of HHb is the negative of ${\mathrm{HbO}}_{2}$ the HPC-RF is infact −1 at all points. This is in line with the hypothesis that brain activation can be expressed as an antiphase relationship between ${\mathrm{HbO}}_{2}$ and HHb.

**Fig. 4:**
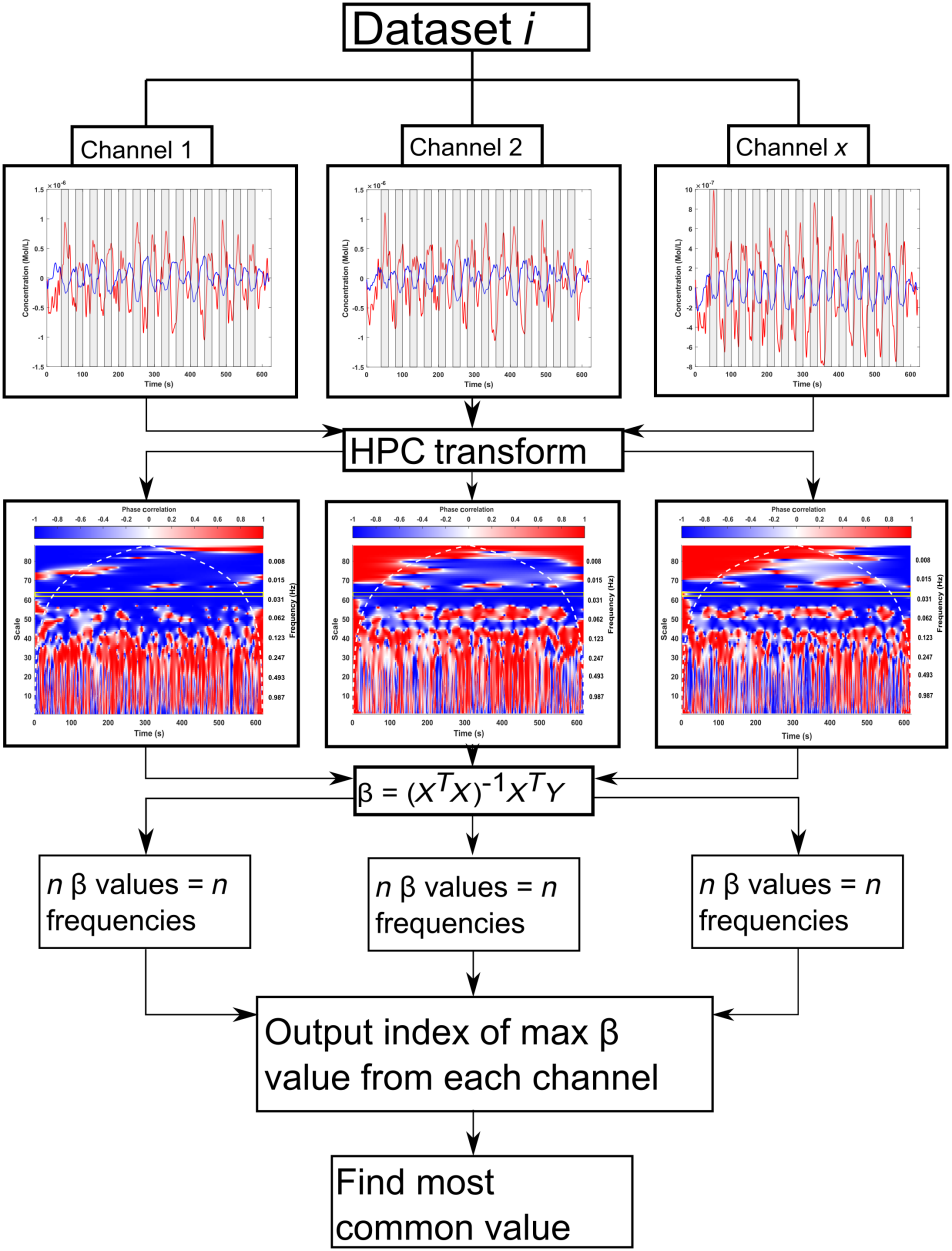
Pipeline used to determine which frequency should be used as the measured signal in the GLM. HPC was computed for all 16 channels of all 18 datasets. The GLM was used to output β values for all frequencies of all HPC transforms. The index of the maximum β value was determined. The index corresponds to the frequency at which that β value was found. The most commonly occurring index was found for each dataset, across channels. The task periods are highlighted in gray in the time data. The outlined area in the HPC spectrograms correspond to frequencies of 0.0234, 0.0250, and 0.0268 Hz. All three are outlined to account for the indirect scale to frequency conversion. The corresponding task-frequency is highlighted in yellow for the HPC spectrograms.

As the HPC exists in the time-domain frequency, a frequency must be chosen to be used in the GLM as the measured signal. For this paper, since both synthetic and experimental data was determined using a constant-frequency block-design task, we hypothesized that the HPC frequency that should be used would correspond to the task frequency used, i.e., for the synthetic data, it would be at 0.025 Hz. This was confirmed using the process shown in Fig. 4.

Due to the nature of the dynamic brain system, we would expect there to be an underlying, base-level of antiphase correlations in the experimental data arising from noise and confounding factors. The effect of this noise on the results would manifest as widespread activations. To account for this base-level of antiphase correlation an activation threshold was determined and used for the experimental data analysis. The threshold would say that a certain level of anticorrelation is required to be reached before considering it to be activation, which should be above 0. It was determined in the β space and represents the lowest value which could be activation. This is possible since the HPC has defined bounds, +1 to −1, and so a specific level of activation can be defined. To decide this threshold, β values were computed from the synthetic data with added activation. Group statistics were determined and signals showing significant activation at p≤0.01 were considered. Of these considered signals, the smallest value was chosen as the activation threshold. The determined threshold value was used as the hypothesized mean for computing t-values using the experimental data.

**Fig. 5:**
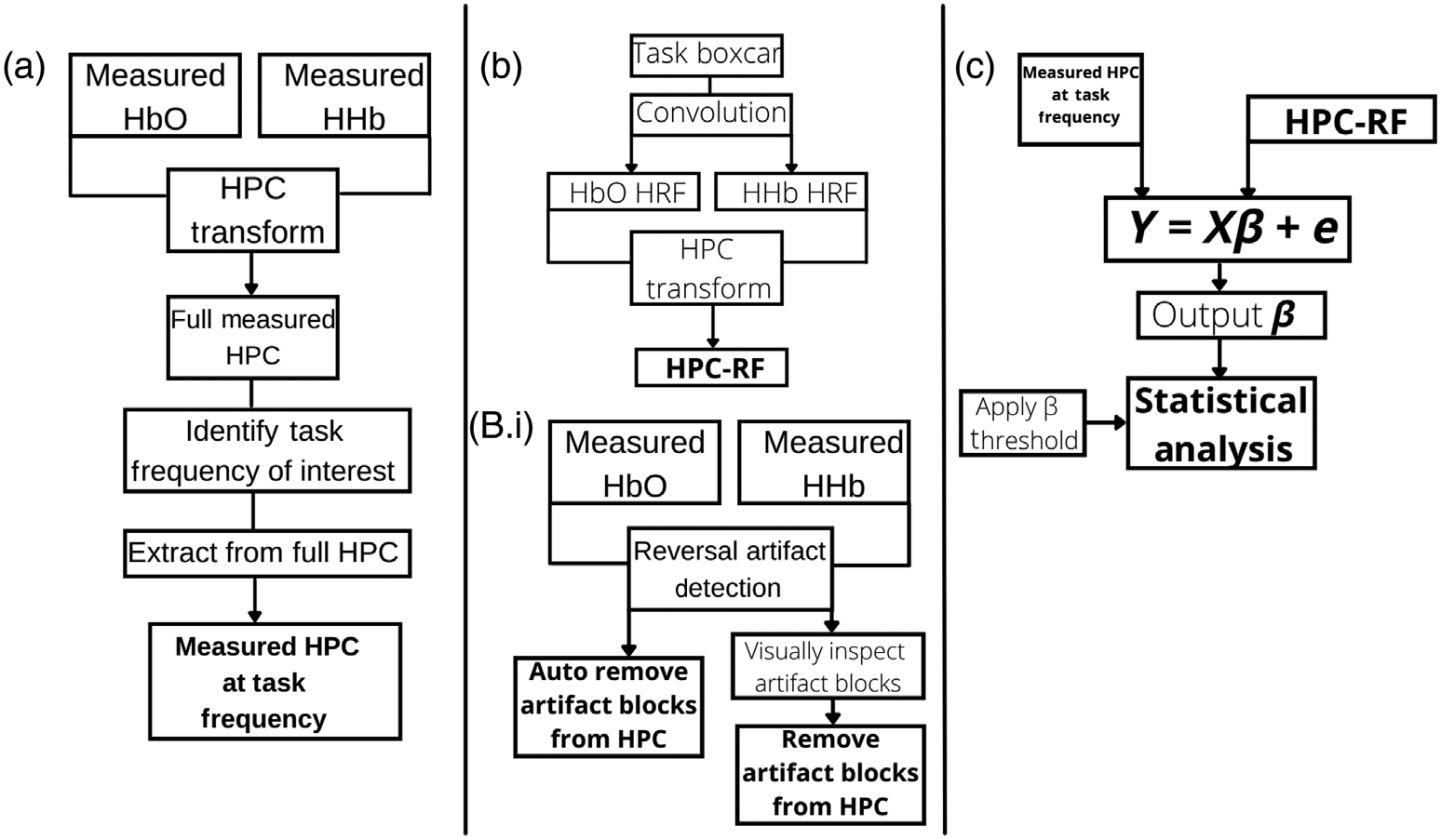
Full workflow of analysis using HPC signal. (a) The HPC is computed using the preprocessed measured ${\mathrm{HbO}}_{2}$ and HHb signals for a given channel. The relevant task frequency is identified, and the HPC signal according to that frequency (or scale) is extracted from the full spectrogram. (b) To compute the HPC-RF the standard HRF of ${\mathrm{HbO}}_{2}$ and HHb are first computed using the task timings, the HPC transform is then applied using the standard HRFs as inputs. (B.i) The reversal artifact detection function can be used at this stage to evaluate blocks that may contain the reversed artifact. Users have the option to automatically remove these blocks from the HPC transform, or to visually inspect blocks using the output as a guide and then remove from analysis. (c) The standard GLM is used with the Measured HPC at a task frequency as the measured parameter (Y in GLM) and the HPC-RF as the input to the design matrix (X in GLM). Once the β values are computed standard statistical analysis can be carried out, and a threshold β value can be applied, the authors suggest a value of $\beta_{\text{Thresh}}\geq 0.3$.

The full workflow using the HPC signal is shown in Fig. 5. The development of the HPC framework was verified using the synthetic data and the results are shown in Sec. 3.1.

### 2.5 Performance Evaluation

The beta values from the synthetic and experimental data were normalized to t-values calculated using Eq. (2). Where x^ refers to the sample mean, μ is a hypothesized mean, σ is the standard deviation and n is the sample size

t=x^−μσn. (2)

For the conventional signals in both synthetic and experimental data, a one-sample, two-tailed t-test was carried out using a hypothesized mean of 0.

The synthetic data performance was evaluated on how large the calculated t-value was for each signal. For the synthetic HPC data, the hypothesized mean was set to 0.

For the experimental data, performance was based on how well a signal could localize activation to the motor cortex region of the brain. To do this, the active conditions of the task were modeled, and β values were obtained for all channels of each participant. For the conventional signals, the task-timings were used to form a task-boxcar function and convolved with the relevant HRF to form the task-related regressor modeling the hemodynamics response. For the HPC signal, the HPC-RF discussed in Sec. 2.4.1 was used, which does not require task timings since the task frequency is known. T-values were then computed based on β values from the GLM using Eq. (2). For the computation of the t-values for the experimental data HPC signal, a one-sample one-tailed t-test was used, and the hypothesized mean was set to the determined activation threshold, which was informed by the synthetic data analysis. Multiple comparisons correction was carried out using the false discovery rate (FDR), using the method outlined in Ref. 86.

### 2.6 Synthetic Data Experiments

Three experiments were conducted with the synthetic dataset to evaluate the performance of each signal. The first experiment aimed at evaluating the performance of each signal in localizing functional brain activity at different SNRs. To this goal, synthetic data were generated with three different amplitudes to provide datasets with varying SNR.

The additional two experiments involved evaluating signals’ performance when activation was removed from signals by reversing the sign of the ${\mathrm{HbO}}_{2}$ and then the HHb task-HRF. This was done to remove the anticorrelated characteristic typical of brain activation and to understand how each signal reflects nonactivation depending on which chromophore (${\mathrm{HbO}}_{2}$ or HHb) is affected. The synthetic data was adapted for this purpose by reversing the polarity of the task-related HRF, in the first case the ${\mathrm{HbO}}_{2}$ was made negative and in the second case, the HHb was made positive. In both cases, this was done incrementally to accentuate the trending behavior of all signals when the polarity is reversed.

## 3 Results

### 3.1 HPC Specific Data Analysis

**Fig. 6:**
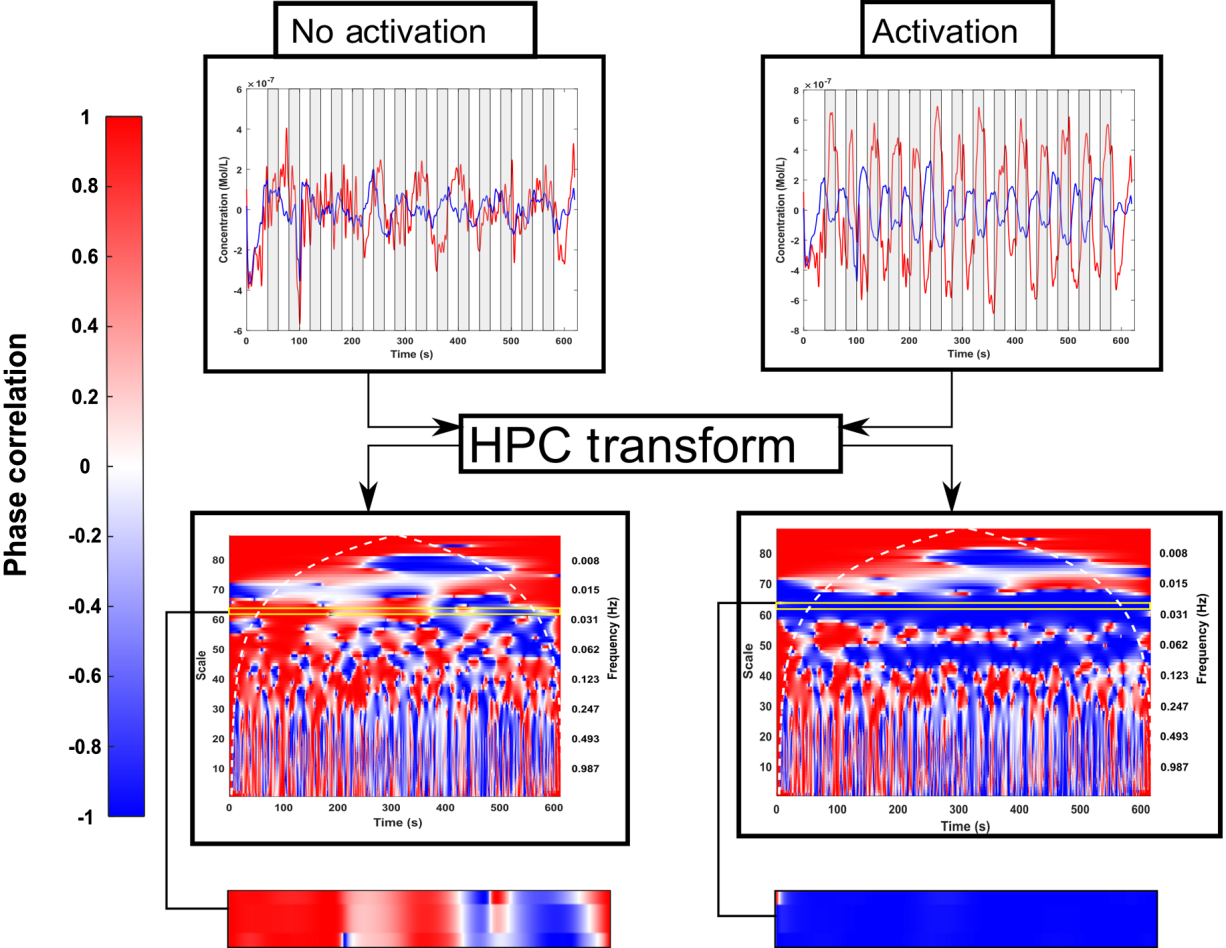
Example HPC output. The plots on the left show the resting state fNIRS data (see Fig. 1) and the corresponding HPC signal. The right shows the same data after the resting state fNIRS data has been added to the synthetic task HRFs with a task frequency of 0.025 Hz. In both HPC, the task frequency is highlighted by a yellow box and zoomed in below. The time data also show the task blocks shaded in gray.

An example of the spectrogram corresponding to the HPC of ${\mathrm{HbO}}_{2}$ and HHb is shown in Fig. 6, using the synthetic data from dataset 3, channel 11. A clear anticorrelation can be seen at the 0.025 Hz task frequency when the activation is encoded into the time series, highlighted by the yellow box on each spectrogram.

**Fig. 7:**
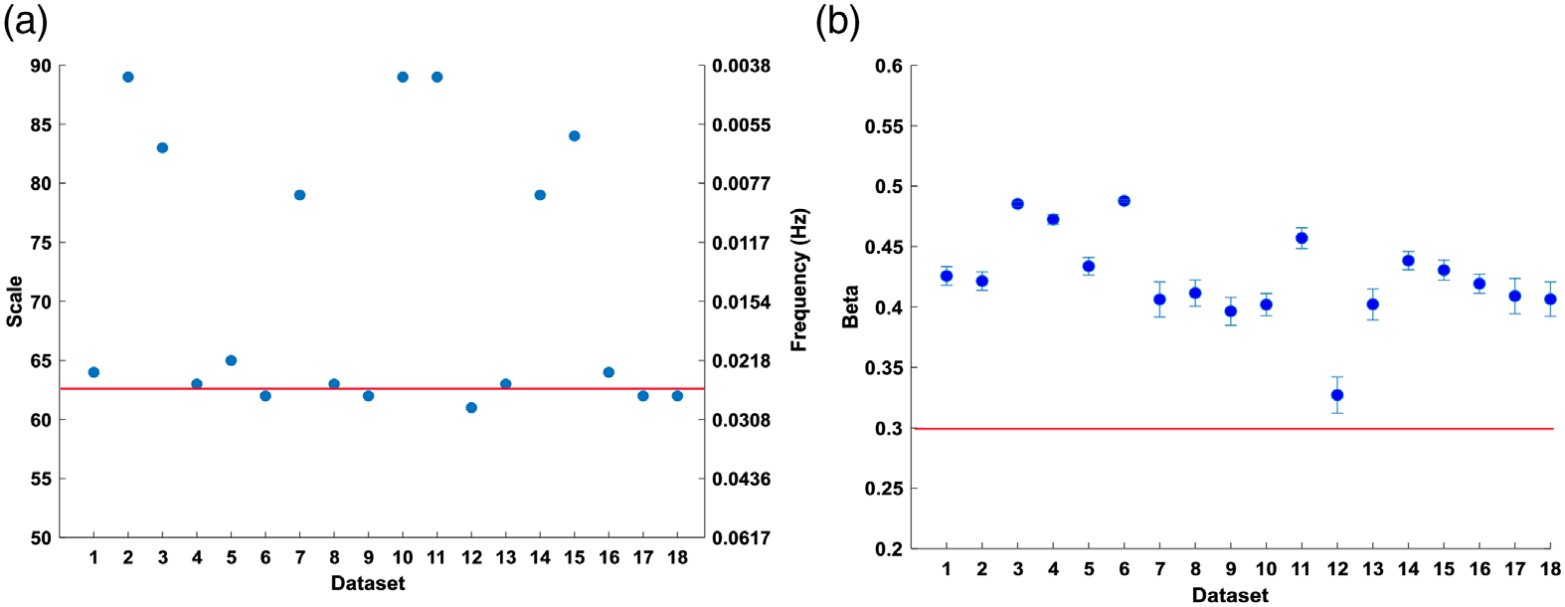
(a) Frequency check results. (b) Activation threshold results; the points correspond to the means as explained in Fig. 4, and the error-bars represent standard error of the mean. The red line in (a) displays the expected task related frequency (0.025 Hz), while (b) displays the chosen threshold based on the results.

The first set of results is from the HPC-specific analysis described in Sec. 2.4.1. The frequency component that should be used in the analysis of the HPC signal is shown in Fig. 7(a) and the evaluation of the HPC-based activation threshold is included in Fig. 7(b).

To determine which frequency from the HPC signal to use the synthetic data with task-component occurring at 0.025 Hz was used. This frequency is shown in Fig. 7(a) by the red line. The frequencies between 0.022 and 0.031 Hz displayed the best fit for 61% of signals. This provided a quantitative basis to determine which frequencies to use in the GLM for the HPC analysis in all following experiments. To account for the discrepancies present in scale-frequency conversion, the frequency present in the HPC closest to the task frequency (3 d.p.) was chosen, the frequency immediately preceding and following was also chosen and the three were averaged together.

To determine the threshold β, the minimum β value from the GLM analysis on the synthetic data was chosen. As activation was encoded in all channels of all datasets the minimum β value found would correspond to the minimum value that activation can occur at, while taking noise into account. To ensure that the activation was present and determinable while including the noise, the beta values from the ${\mathrm{HbO}}_{2}$ and the HHb signals were tested for significance at p<0.01 and the minimum β value of the channels showing significance was chosen as the threshold. The minimum value found was 0.32, to account for variations the threshold was chosen to be 0.3, shown by the red line in Fig. 7(b). This value would be used as the hypothesized mean of the experimental data.

### 3.2 Synthetic Data

**Fig. 8:**
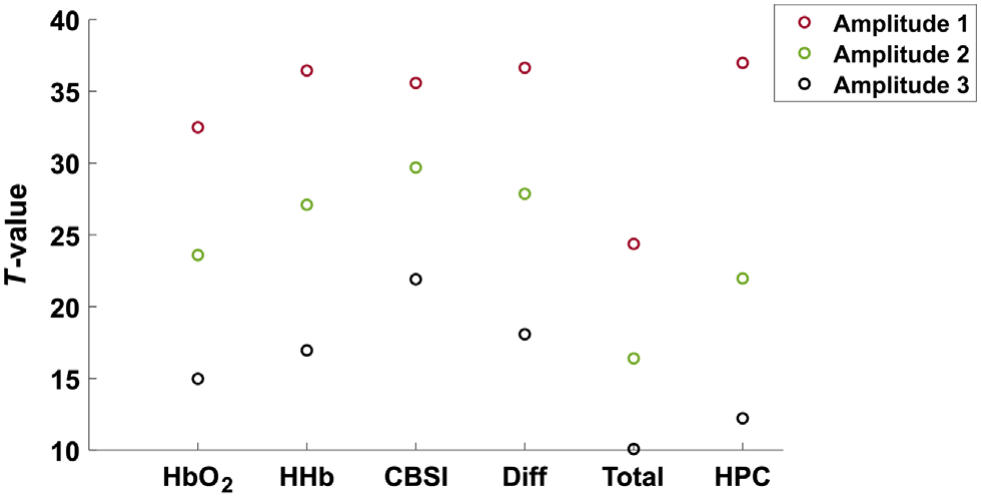
Percentage deviation of all signals when varying the amplitude of the task-related component of the synthetic data. Amplitude 1 has the highest signal-noise ratio. T-values were computed using a one-sample two-tailed t-test, with a hypothesized mean of 0.

**Fig. 9:**
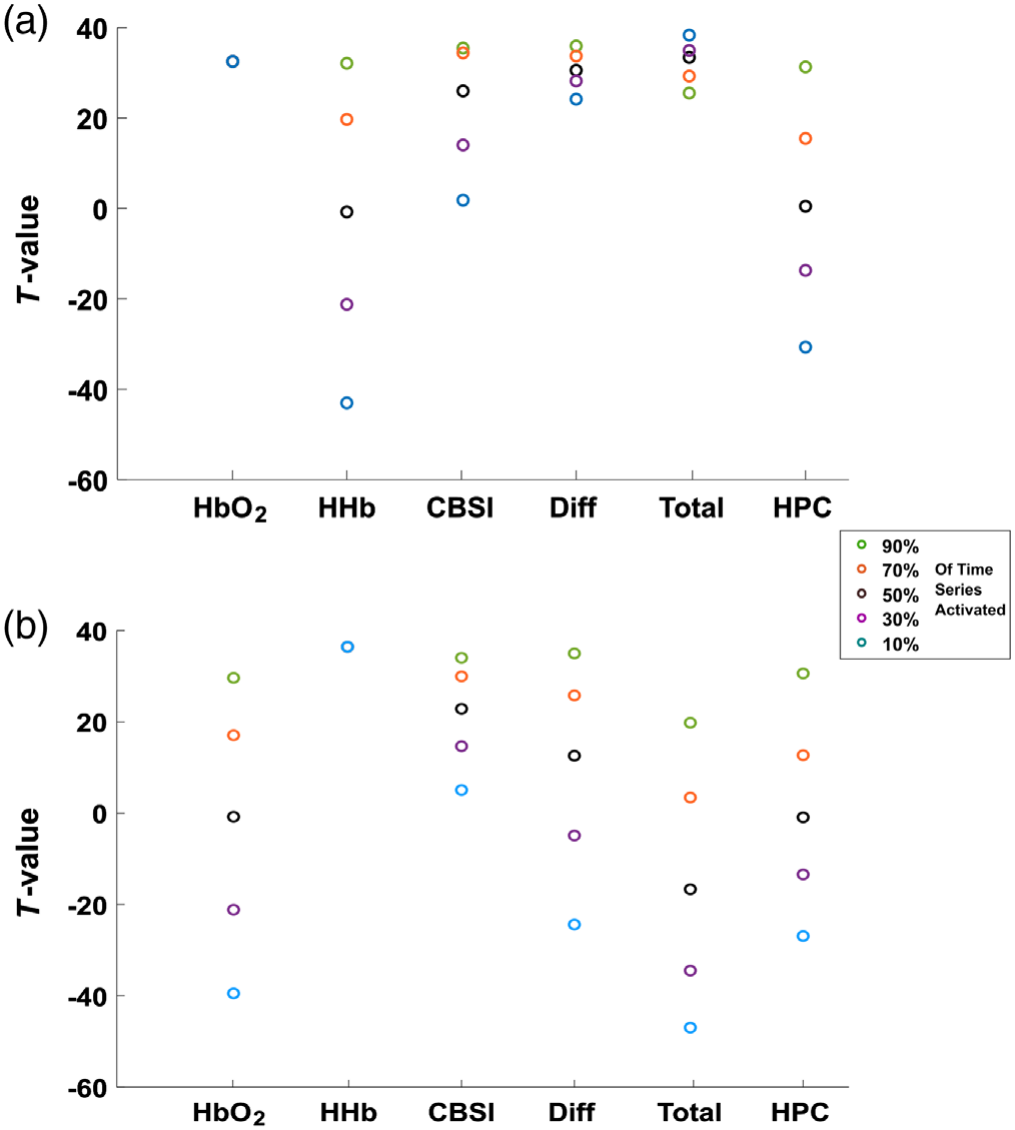
(a) Results from reversing the sign of the HHb HRF and (b) results from reversing the sign of the ${\mathrm{HbO}}_{2}$ signal. The percentages listed in the legend refer to what percentage of the time series was convolved with a task-related HRF, 90% corresponds to near full activation. T-values were computed using a one-sample two-tailed t-test with hypothesized mean equal to 0.

Figures 8 and 9 display the results of the experiments conducted using the synthetic dataset. For both Figs. 8 and 9, the raw beta values can be seen in the Supplemental Material (Fig. S13 in the Supplemental Material). In all cases, a higher t-value indicates a signal which has a closer fit with the task-related HRF used in the GLM. Figure 8 shows the results for varying SNR. Amplitude 3 is the signal with the lowest SNR. For all signals, the t-values decrease as SNR reduces, as expected. Table 3 shows the numerical values for Fig. 8. The legend entries in Figs. 9(a) and 9(b) refer to how much of the time series is encoded with activation. At 10% only 10% of the time series is encoded with activation by a positive ${\mathrm{HbO}}_{2}$ and a negative HHb. The SNR used for these tests was amplitude 1, as such the results for 90% relate closely to the results for amplitude 1 in Fig. 8. The results when HHb HRF is made positive are shown in Fig. 9(a), and when ${\mathrm{HbO}}_{2}$ is negative in Fig. 9(b).

**Table 3:** Numeric t-values for Fig. 8.

	${\mathrm{HbO}}_{2}$	HHb	CBSI	${\mathrm{Hb}}_{\mathrm{Diff}}$	${\mathrm{Hb}}_{\mathrm{Tot}}$	HPC
Amp 1	32.4	36.4	35.6	36.6	24.4	36.9
Amp 2	23.6	27.1	29.7	27.9	16.4	21.9
Amp 3	14.9	16.9	21.9	18.1	10.1	12.2

The ${\mathrm{HbO}}_{2}$ result in Fig. 9(a) is constant at 32.4, expected as the ${\mathrm{HbO}}_{2}$ data are not altered. Comparatively, the HHb signal ranges from 32.1 at 90% to −43 at 10%, representing the intended effect of reversing the sign of HHb. CBSI displays similar behavior, t-values ranging from 35.5 at 10% to 1.8 at 90%. ${\mathrm{Hb}}_{\text{Diff}}$ ranges from 35.9 to 24.2. ${\mathrm{Hb}}_{\text{Total}}$ displayed the opposite effect, at 10% the t-value was 38.3, while at 90% it was 25.5. Finally, the HPC ranges from 31.3 to −30.7.

As expected for Fig. 9(b), the HHb response is constant at 36.4, as the data for HHb were not altered. The ${\mathrm{HbO}}_{2}$ ranges from 29.6 at 90% activation to −39.5. CBSI displays the same pattern as shown in Fig. 9(a), ranging from 34.0 at 90% activation to 5.1 at 10% activation. ${\mathrm{Hb}}_{\mathrm{Diff}}$ ranges from 34.98 to −24.39. ${\mathrm{Hb}}_{\text{Total}}$ ranges from 19.8 to −46.9. Finally, the HPC signal ranges from 30.6 to −26.9, showing the same pattern as Fig. 9(b).

### 3.3 Experimental Data

**Fig. 10:**
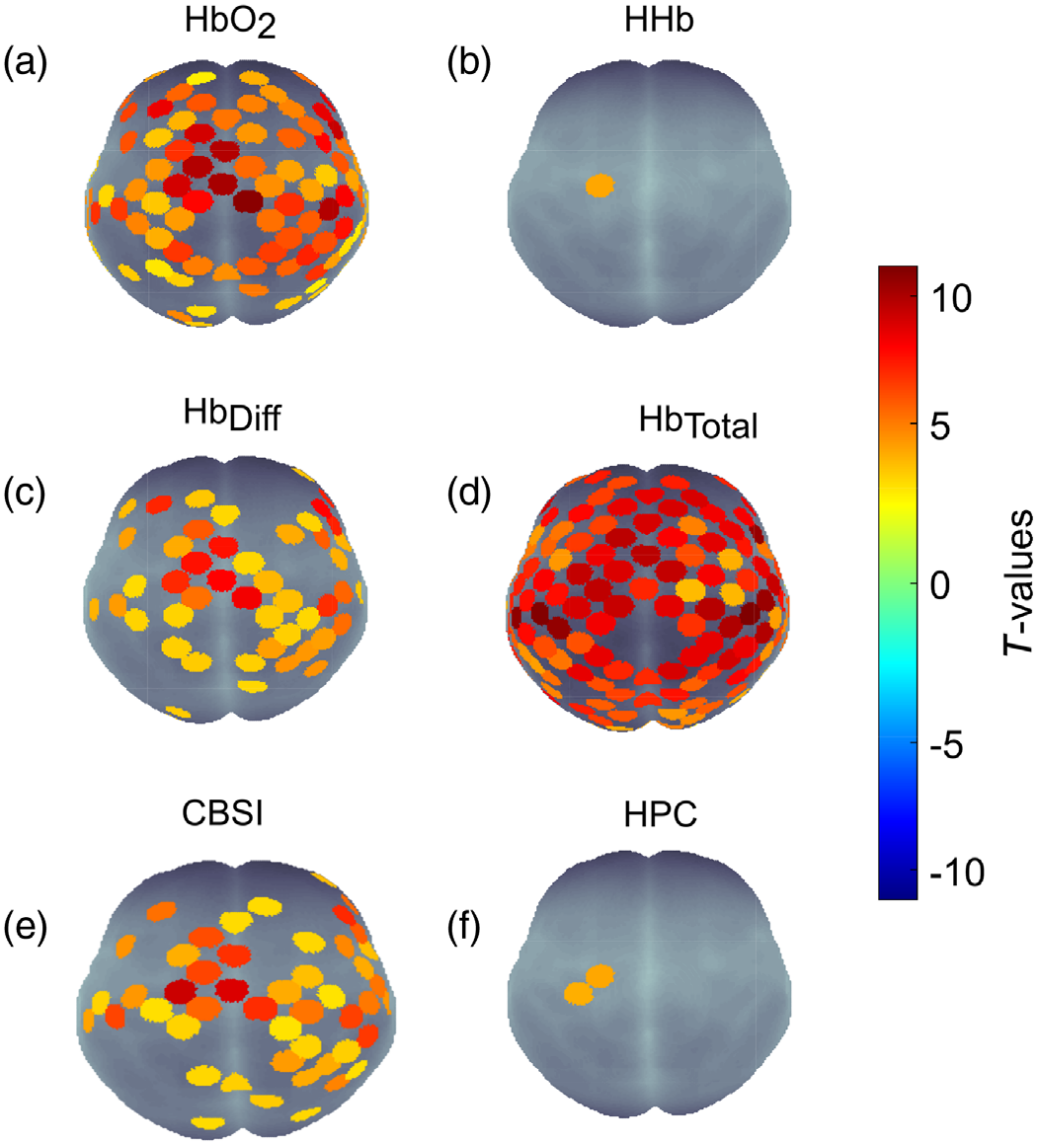
All signals, FDR corrected p-value≤0.01. (a) ${\mathrm{HbO}}_{2}$, (b) HHb, (c) ${\mathrm{Hb}}_{\mathrm{Diff}}$, (d) ${\mathrm{Hb}}_{\text{Total}}$, (e) CBSI, and (f) HPC. The experimental task was a right-handed finger-thumb tapping task. Expected channel activations were in the left primary motor cortex.

The group results for the finger-thumb tapping task are shown in Fig. 10. The views shown are axial view, where the top of each image would be the front of the body. The p-value threshold was set at p≤0.01 after applying FDR correction.

The ${\mathrm{HbO}}_{2}$ signal found 96 channels with activation. The mean t-score across these channels was 5.13 (σ=±0.176) and the mean p-value $9.28\times 10^{-04}$ ($\sigma =\pm 2.11\times 10^{-04}$). In contrast, HHb showed 1 channel with activation with a t-score of 4.18 and p-value of 0.007, localized to the left primary motor cortex. CBSI and ${\mathrm{Hb}}_{\mathrm{Diff}}$ found 48 channels being active, in slightly different locations. CBSI had a mean t-score of 4.56 (σ=±0.200) compared with ${\mathrm{Hb}}_{\mathrm{Diff}}$ with 4.60 (σ=±0.207). Similarly, CBSI had mean p-value of 0.002 ($\sigma =\pm 4.03\times 10^{-04}$) compared with ${\mathrm{Hb}}_{\mathrm{Diff}}$ with p-value 0.0015 ($\sigma =\pm 2.541\times 10^{-04}$). ${\mathrm{Hb}}_{\text{Total}}$ showed the most activated channels, with 131 below the p-value threshold, with mean p-value of $1.80\times 10^{-04}$ ($\sigma =\pm 9.89\times 10^{-05}$) and mean t-score at 6.55 (σ=±0.144). Finally, the HPC displayed similar performance to HHb showing two activated channels with a mean p-value 0.0035 and mean t-score 4.10 (σ=±0.092).

## 4 Discussion

The HPC signal is formed based on the fundamental characteristic of antiphase correlation between ${\mathrm{HbO}}_{2}$ and HHb present in the hemodynamic response. Thus, the signal is encoded with information from both ${\mathrm{HbO}}_{2}$ and HHb. This key design aspect has two benefits: first, by incorporating information from both chromophores we improve the statistical robustness of the results; second, we reduce confusion in the field in how to analyze and interpret both signals by combining them into one signal.

We have first assessed the performance of ${\mathrm{HbO}}_{2}$, HHb, CBSI, ${\mathrm{Hb}}_{\mathrm{Diff}}$, ${\mathrm{Hb}}_{\text{Total}}$, and HPC using synthetic data. The results are shown in Figs. 8 and 9. The first test (Fig. 8) showed that the CBSI signal performed the most consistently, with the highest t-values for amplitudes 2 and 3, but slightly worse than HHb, ${\mathrm{Hb}}_{\mathrm{Diff}}$, and HPC for amplitude 1. This result suggests that the CBSI is the most robust against a reducing SNR since it is the least affected by the decreasing SNR from amplitudes 2 and 3.

The HPC signal provided the highest t-value when the SNR was high, suggesting that the HPC is able to indicate brain activation. However, the HPC seems to be the most susceptible to the decreasing SNR, indicated by a large fall in t-value from amplitude 1 to 2. By decreasing the amplitudes of ${\mathrm{HbO}}_{2}$ and HHb the noise in the signal will dominate and dilute the antiphase relationship between ${\mathrm{HbO}}_{2}$ and HHb. This result was the driving force behind the requirement for an activation threshold.

We have also carried out an analysis with the synthetic data by forcibly introducing false positives into the time series data in order to further demonstrate that the use of a single chromophore is not robust enough against false positives which may arise due to systemic physiology, artifacts, or otherwise. The results are shown in Figs. 9(a) and 9(b). The ideal signal, in this case, would reflect the degree of activation and perform uniformly, independent of whether it was ${\mathrm{HbO}}_{2}$ or HHb that was altered. In this test, the signals which were formed using the anticorrelation present in brain activation (CBSI, HPC) achieved both of these requirements. Since they are encoded with the specific characteristic of the relationship between ${\mathrm{HbO}}_{2}$ and HHb, it seems they are better suited to determine activation based on a GLM fit. An interesting point however is that although the CBSI does determine low activation, the HPC is able to determine an “inverse” activation, where it can determine that either ${\mathrm{HbO}}_{2}$ or HHb are in the opposite polarity. In comparison, the two single chromophores were unable to distinguish false positives. Similarly, the effect on a single chromophore had repercussions on the t-value of ${\mathrm{Hb}}_{\text{Total}}$ and ${\mathrm{Hb}}_{\mathrm{Diff}}$ depending on which chromophore was altered. ${\mathrm{Hb}}_{\text{Total}}$ performed the worst of all the signals, in the case where HHb was altered ${\mathrm{Hb}}_{\text{Total}}$ displayed the opposite of the expected trend; at low activation, it showed a high t-value. This is due to the HHb signal becoming positive and, therefore, the formation leads to a larger signal. Similarly, ${\mathrm{Hb}}_{\mathrm{Diff}}$ was affected differently depending on the chromophore being altered however it displayed the expected trend of high t-value at high activation and decreasing t-value with activation.

The difference between the CBSI, HPC and ${\mathrm{Hb}}_{\mathrm{Diff}}$, ${\mathrm{Hb}}_{\text{Total}}$ stands in their formation. CBSI and HPC are derived on the basis of a physiological characteristic of brain activation (i.e., anticorrelated patterns of ${\mathrm{HbO}}_{2}$ and HHb). Thus, as long as that specific characteristic is present in the fNIRS data, the CBSI and HPC are better suited to detect brain activation.

To our knowledge, this is the first study that has evaluated and compared the performance of the most commonly used signals in the field using the GLM approach. The results from the synthetic analysis suggest that the use of a signal formed from the combination of ${\mathrm{HbO}}_{2}$ and HHb is more robust against a decreasing SNR and the possibility of false positives in the time series. As shown by either the HPC or CBSI performing the best when the SNR was varied and also being able to uniformly and adequately reflect the degree of activation in the following two tests. This method of deriving functional activation from the combination of ${\mathrm{HbO}}_{2}$ and HHb was mentioned in Ref. 9, where the authors specifically cited the CBSI method as a way to derive functional activation based on a specific characteristic of activation, such as the anticorrelation used in the CBSI or the antiphase correlation used by the HPC. Furthermore, both the HPC and the CBSI satisfy the crucial requirement to use both ${\mathrm{HbO}}_{2}$ and HHb as stated by the fNIRS best practices^10^ as it is encoded with information from both ${\mathrm{HbO}}_{2}$ and HHb. The HHb signal performed third best in all amplitudes, better than the ${\mathrm{HbO}}_{2}$ signal, possibly because it is less susceptible to the systemic noise component as suggested by previous studies.^3^^,^^8^

We next assessed the signals using fNIRS data acquired from a finger-tapping task. Data were acquired from 128 adult participants with 134 channels covering the entire head. Signals’ performance was evaluated on their ability to localize activation with respect to the target anatomical location (ground truth). Here our target region was the left motor cortex for a right finger tapping task. As shown in Fig. 10, we found that the HHb signal provided the best localization with a single channel localized over the Precentral Gyrus, the site of the primary motor cortex. However, the t-value was lower than the corresponding channel for ${\mathrm{HbO}}_{2}$, suggesting HHb has reduced statistical power compared with ${\mathrm{HbO}}_{2}$. This region has been implicated in fMRI and PET studies^87^^–^^91^ as well as in fNIRS studies.^92^^–^^95^ More specifically,^93^ also deduced that the HHb signal provided more localized activation than the ${\mathrm{HbO}}_{2}$ signal in a motor task, in agreement with our findings. Studies have shown the HHb signal is more highly correlated to the BOLD signal than ${\mathrm{HbO}}_{2}$,^19^ therefore, it was expected for the HHb signal to display results in agreement with the fMRI BOLD signal. However, as we have shown with the synthetic data a single chromophore should not be used to make interpretations due to the possibility of false positives and negatives, and results obtained from one signal should be looked at with caution. A further analysis (shown in Fig. S15 in the Supplemental Material) of this dataset showed the HHb signal was positively increasing alongside ${\mathrm{HbO}}_{2}$ for a widespread number of channels. HHb increasing with ${\mathrm{HbO}}_{2}$ is a typical characteristic of a systemic change.^2^^,^^7^ This manifested in a larger ${\mathrm{Hb}}_{\text{Total}}$ increase than expected, represented in the results as a widespread ${\mathrm{Hb}}_{\text{Total}}$ false-positive activation. These results show how the possibility of false-positives in a single chromophore can affect the statistical inference of activation. The HPC signal was able to provide more specific localization than the other signals formed from ${\mathrm{HbO}}_{2}$ and HHb (i.e., CBSI, ${\mathrm{Hb}}_{\mathrm{Diff}}$, and ${\mathrm{Hb}}_{\text{Total}}$). The two channels that displayed significant activation correspond to the Precentral Gyrus, the same as the HHb signal. The HPC offers increased confidence in the results because it is based on the fundamental antiphase relationship intrinsic to the hemodynamic response. The CBSI and ${\mathrm{Hb}}_{\mathrm{Diff}}$ signals displayed 38 channels in common showing activation, with 10 different. CBSI showed one more channel located in the left motor cortex that was not present in the ${\mathrm{Hb}}_{\mathrm{Diff}}$ signal. The similarity between these two signals is due to the formula used to form the CBSI, (see Ref. 56). They differ in that the CBSI signal has a parameter (α), formed by the ratio of the standard deviations of the measured ${\mathrm{HbO}}_{2}$ and HHb signals across the whole time series for that channel. This parameter reflects the ratio of the standard deviation of ${\mathrm{HbO}}_{2}$ and HHb, representative of the ratio of the noise amplitude in the signal. The similarity between the CBSI and ${\mathrm{Hb}}_{\mathrm{Diff}}$ was also shown in the synthetic data SNR test, where the ${\mathrm{Hb}}_{\mathrm{Diff}}$ was one position lower than the CBSI in amplitudes 2 and 3 and within a t-value of 1 for amplitude 1.

In comparison to the ${\mathrm{Hb}}_{\mathrm{Diff}}$, ${\mathrm{Hb}}_{\text{Total}}$, and the CBSI signals, since the HPC does not consider the amplitude of ${\mathrm{HbO}}_{2}$ and HHb in its formation, it is not susceptible to large increases due to false positives or negatives in its constituent signals or to unexpected noise amplitudes. We believe that its sensitivity to the fundamental antiphase of ${\mathrm{HbO}}_{2}$ and HHb has prevented it from detecting the channels where ${\mathrm{HbO}}_{2}$ and HHb were increasing or decreasing simultaneously.

Taken together, our results on synthetic and experimental data suggest that the HPC signal has the potential for the identification of functional brain activity from fNIRS data and may contribute to increased robustness against false positives and negatives. Considering the individual signals only, the results suggest the HHb signal is better able to localize brain activation compared with the ${\mathrm{HbO}}_{2}$ signal.

This paper did not evaluate the performance of any signals after other signal processing methods were applied. It has been noted that the acquisition of fNIRS data from short channels during data acquisition and subsequent usage as a regressor in the GLM can significantly improve the ${\mathrm{HbO}}_{2}$ results by essentially removing the systemic components from the analysis.^96^ Software-based methods such as the Spatial filter^97^ also provide methods of improving the individual signals acquired from the fNIRS system. Therefore, it is possible to obtain good fNIRS data while using the ${\mathrm{HbO}}_{2}$ and HHb signals separately. The HPC signal offers the advantage of providing adequate localization without the added complexity of physically incorporating short channels, the reduced spatial resolution of dedicating optodes to short channels, or the increased analytic complexity of using short-channel data or additional software-based signal processing methods.

In our future studies, we plan to validate the HPC analysis further using more diverse task-protocols to examine how more severe systemic influences affect the HPC, as well as develop the method to be used with varying task-frequencies and conditions occurring at the same frequency. The threshold values specified in this paper can be used as a default value for initial use, however other users can tweak the value as desired, similarly for the “n” parameter in Eq. (4), Fig. 3 we suggest a default value of 1. Further work will also be conducted to optimize the detection of reversed activation response patterns and to minimize the impact of confounding factors on the HPC-based localization of functional brain activity. In our future experiments, we will investigate and refine these values further. Furthermore, we believe the HPC can be used to determine channel quality by numerically presenting the relationship between ${\mathrm{HbO}}_{2}$ and HHb. For example, if there is a large systemic influence, we will see a concurrent increase in ${\mathrm{HbO}}_{2}$ and HHb, which the HPC will reflect. Finally, version 1.0 of the toolbox is under development which will provide all the functionality of the HPC signal in an easy-to-use graphical user interface, allowing integration with popular analysis toolboxes.

## 5 Conclusion

We introduced a new combined signal and evaluates the performance of inferring activation against other signals. Among these, the HHb signal resulted to be the single individual chromophore with the highest accuracy in the identification of brain activation. However, our results further suggested that using one chromophore as a means to make interpretations of functional activation might not be adequate to account for false positives and/or false negatives. Our initial investigation on the HPC signal provided promising results, showing comparable performance to HHb but going beyond that by incorporating information from ${\mathrm{HbO}}_{2}$, hence being less prone to false positives and/or negatives.

The evidence provided in this work can have a significant impact in the fNIRS field. The HPC signal is able to localize activation and encodes information from ${\mathrm{HbO}}_{2}$ and HHb. The comparison of signals will help to reduce the heterogeneity and uncertainty around the analysis and interpretation of ${\mathrm{HbO}}_{2}$ and HHb.

## Code, Data, and Materials Availability

The code for the HPC v1.0 and sample data are available at https://github.com/multimodalspectroscopy/HPC.
