# Supplementary material for: Investigation of functional near-infrared spectroscopy signal quality and development of the hemodynamic phase correlation signal
Source: Neurophotonics. 2022 May 18;9(2):025001. doi: 10.1117/1.NPh.9.2.025001 (PMC9116886; doi:10.1117/1.NPh.9.2.025001)
Supplement: Supplementary file 1 [file NPh_009_025001_SD001.pdf]

Supplementary Materials

Reviewed Papers

Title	First Author	Year	Signal
Effects of aging on prefrontal brain activation during challenging walking conditions	Mirelman, A	2017	CBSI
Investigating the role of temporal lobe activation in speech perception accuracy with normal hearing adults: An event-related fNIRS study	Defenderfer, J	2017	HbO2 and HbR
fNIRS assessment of reward perception based on visual self-expression: Coloring, doodling and free drawing	Kaimal, G	2017	HbO2
Prefrontal cortical responses in children with prenatal alcohol-related neurodevelopmental impairment: A fNIRS study	Kable, J	2017	HbO2 and HbR
Impact of associative word learning on phonotactic processing in 6-month old infants: A combined EEG and fNIRS study	Obrig, H	2017	HbO2 and HbR
Discrimination of emotional prosodies in human neonates: A pilot fNIRS study	Zhang, D	2017	HbO2
Frontal activity during a verbal emotional working memory task in patients with Alzheimers Disease: A fNIRS study	Ates, F	2017	HbO2
Motor planning and performance in trasitive and intransitive gesture execution and imagination: Does EEG (RP) activity predict hemodynamic (fNIRS) response?	Balconi, M	2017	HbO2 and HbR
Using fNIRS to examine occipital and temporal responses to stimulus repetition in young infants: Evidence of slective frontal cortex involvement	Emberson, L	2017	HbO2
Classification of somatosensory cortex activities using fNIRS	Hong, KS	2017	HbO2
Social risky-decision-making reveals gender differences in the TPJ: A hyperscanning study using fNIRS	Zhang, M	2017	HbO2
Decreased prefrontal brain activation during verbal fluency task in patients with somatoform pain disorder: An exploratory multi-channel NIRS study	Ren, X	2017	HbO2
Neural correlates of decision making on whole body yaw rotation: An fNIRS study	de Winkel, KN	2017	HbT
Functional Brain Imaging of Walking While Talkng	Metzger, F	2017	CBSI
Brain activity underlying the recovery of meaning from degraded speech: A fNIRS study	Wijayasiri, P	2017	Other
Relevance of Dorsolateral and Frontotemporal Cortex on the Phonemic Verbal Fluency	Herrmann, M	2017	HbO2 and HbR
Force related haemodynamic responses during execution and imagery of a hand grip task: A fNIRS study	Wriessnegger, SC	2017	HbO2 and HbR
Frontal temporal and parietal systems synchronize within and across brains during live eye-to-eye contact	Hirsch, J	2017	HbR
Prefrontal cortex activation during obstacle negotiation: What's the effect size and timing?	Maidan, I	2018	HbO2
Detecting concealed information with fused electroencephalograpy and fNIRS	Lin, X	2018	HbO2
Dynamic causal modelling on infant fNIRS data: A validation study on a simultaneously recorded fNIRS-fMRI dataset	Bulgarelli, C	2018	HbO2
Reduced motor cortex inhibition and a 'cognitive-first' prioritisation strategy for older adults during dual-tasking	Corp, D	2018	Hdiff

Comparison of virtual reality and hands on activities in science education via fNIRS	Lamb, R	2018	HbO2
The relationship between the superior frontal cortex and alpha oscillation in a flanker task: Simultaneous recording of EEG and NIRS	Suzuki, K	2018	Other
Negative affect is related to reduced differential neural responses to social and non-social stimuli in 5-to-8 month old infants: A fNIRS study	Van Der Kant, A	2018	HbO2 and HbR
Cooperation makes two less-creative individuals turn into a highly-creative pair	Xue, H	2018	HbO2
Brain-to-brain synchrony in parent child dyads and the relationship with emotion regulation revealed by fNIRS-based hyperscanning	Reindl, V	2018	HbO2
Interpersonal brain synchronization associated with working alliance during psychological counselling	Zhang, Y	2018	HbO2
A transferable high-intensity intermittent exercise improves executive performance in association with dorsolateral prefrontal activation in young adults	Kujach, S	2018	HbO2 and HbR
Neural basis for reduced executive performance with hypoxic exercise	Ochi, G	2018	HbO2
Eye contact modulates facial mimicry in 4-month-old infants: An EMG and fNIRS study	de Klerk, CCJM	2018	HbO2 and HbR
Changes in cerebral activation in individuals with and without visual vertigo during optic flow: A fNIRS study	Hoppes, C	2018	HbO2
Relationship between sensorimotor cortical activation as assessed fNIRS and lower extremity motor coordination in bilateral cerebral palsy	Sukal-Moulton, T	2018	HbT
Meditation and the brain - Neuronal correlates of mindfulness as assessed with NIRS	Gundel, F	2018	HbR
Interpersonal synchronization of inferior frontal cortices tracks social interactive learning of a song	Pan, Y	2018	CBSI
Cortical correlates of speech intelligibility measured using fNIRS	Lawrence, RJ	2018	Other
Stress-related dysfunction of the right inferior frontal cortex in high ruminators: An fNIRS study	Rosenbaum, D	2018	CBSI
Cortical hemodynamic changes during the Trier Social Stress Test: An fNIRS study	Rosenbaum, D	2018	CBSI
Modality independent recruitment of ingerior frontal cortex during speech processing in human infants	Altvater-Mackensen, N	2018	HbO2 and HbR
Building predictive models of emotion with fNIRS	Bandara, D	2018	HbO2 and HbR
Forgiveness and cognitive control - Provoking revenge via theta-burst stimulation of the DLPFC	Maier, MJ	2018	CBSI
Decreased haemodynamic response in inferior frontotemporal regions in elderly with mild cognitive impairment	Katzorke, A	2018	HbO2 and HbR
Delayed hemodynamic responses associated with a history of suicide attempts in bipolar disorder: A multichannel NIRS study	Hirose, T	2018	HbO2
Imagine squeezing a cactus: Cortical activation during affective motor imagery measured by fNIRS	Wriessnegger, S	2018	HbO2 and HbR
Cortical activity in fine-motor tasks in children with Developmental Coordination Disorder: A preliminary fNIRS study	Cacola, P	2018	HbO2
The association between prefrontal cortex activity and turning behavior in people with and without freezing of gait	Belluscio, V	2019	HbO2
Cognitive flexibility-related prefrontal activation in preschoolers: A biological approach to temperamental effortful control	Quinones-Camacho, L	2019	HbO2
Decreased prefrontal connectivity parallels cognitive fatigue-related performance decline afer sleep deprivation. An optical imaging study	Borragan, G	2019	Other
Moral incompetency under time constraint	Lee, E	2019	HbO2

Differences in brain signal complexity between experts and novices when solving conceptual science problem: A fNIRS study	Jin, L	2019	HbO2 and HbR
Psychophysiological indices of cognitive style: A triangulated study incorporating neuroimaging, eye-tracking, psychometric and behavioural measures	Bendall, R	2019	HbO2
Enhancing neural efficiency of cognitive processing speed via training and neurostimulation: An fNIRS and TMS study	Curtin, A	2019	HbO2 and HbR
Brain mechanisms for processing discriminative and affective touch in 7-month-old infants	Miguel, H	2019	HbO2
Ready, set, go: Cortical hemodynamics during self-controlled sprint starts	Wolff, W	2019	HbO2
Inter-brain synchrony in mother-child dyads during cooperation: An fNIRS hyperscanning study	Miller, J	2019	HbO2
Brain activation and adaption of deception processing during dyadic face-to-face interaction	Tang, H	2019	HbO2
Differentiation in prefrontal cortex recruitment during childhood: Evidence from cognitive control demands and social contexts	Chevalier, N	2019	HbO2
Shared neural representations of syntax during online dyadic communication	Liu, W	2019	HbO2
Praising or keeping silent on partner's ideas: Leading brainstorming in particular ways	Lu, K	2019	HbO2
Assessing the brain 'on the line': An ecologically-valid assessment of the impact of repetitive assembly line work on hemodynamic response and fine motor control using fNIRS	Han, W	2019	HbO2
Real-life creative problem solving in teams: fNIRS based hyperscanning study	Mayseless, N	2019	HbO2
A combined EEG-fNIRS study investigating mechanisms underlying the association between aerobic fitness and inhibitory control in young adults	Ludyga, S	2019	HbO2
Infant brain responses to social sounds: A longitudinal fNIRS study	McDonald, N	2019	HbO2
Expectation affects neural repetition suppression in infancy	Emberson, L	2019	HbO2
Absence of neural speech discrimination in preterm infants at term-equivalent age	Bartha-Doering, L	2019	HbO2
Hand or spoon? Exploring the neural basis of affective touch in 5-month-old infants	Pirazzoli, L	2019	HbO2 and HbR
Anticipation of a mentally effortful task recruits Dorsolateral Prefrontal Cortex: An fNIRS validation study	Vassena, E	2019	HbO2
Selective facial mimicry of native over foreign speakers in preverbal infants	de Klerk, CCJM	2019	HbO2
Empirical evidence that concept mapping reduces neurocognitive effort during concept generation for sustainability	Hu, M	2019	HbO2
Effective connectivity of the fronto-parietal network during the tangram task in a natural environment	Hu, Z	2019	HbO2
IQ Estimation by means of EEG-fNIRS recordings during a logical-mathematical intelligence test	Firooz, S	2019	Not Stated
A fNIRS investigation of Speech Planning and Execution in Adults who Stutter	Jackson, ES	2019	HbO2 and HbR
Monitoring multiple cortical regions during walking in young and older adults: Dual-task response and comparison chalenges	Stuart, S	2019	HbO2
Arithmetic learning modifies the functional connectivity of the fronto-parietal network	Zhao, H	2019	HbO2
Effects of music therapy on major depressve disorder: A study of prefrontal haemodynamic functions using fNIRS	Feng, K	2019	HbO2
Interplay between prior knowledge and communication mode on teaching effectiveness: Interpersonal neural synchronization as a neural marker	Liu, J	2019	CBSI
How does the embodied metaphor affect creative thinking?	Wang, X	2019	HbO2

Hemispheric mPFC asymmetry in decision making under ambiguity and risk: An fNIRS study	Li, Y	2019	HbO2
The effects of CACNA1C gene polymorphism on prefrontal cortex in both schizophrenia patients and healthy controls	Zhang, Z	2019	HbO2 and HbR
Changes of functional response in sensorimotor cortex of preterm and full-term infants during the first year: An fNIRS study	Rosa de Oliveira, S	2019	HbO2 and HbR
The role of the right prerontal cortex in recognition of facial expressions in depressed individuals: fNIRS study	Mannelis, A	2019	HbR
English spoken word segmentation activates the prefrontal cortex and temporo-parietal junction in Chinese ESL learners: A fNIRS study	Li, Y	2020	HbO2
Infant brain responses to live face-to-face interaction with their mothers: Combining fNIRS with a modified still-face paradigm	Behrendt, H F	2020	HbO2
So young, yet so mature? Electrophysiological and vascular correlates of phonotactic processing in 18-month-olds	Steber, S	2020	HbO2 and HbR
Evaluation of neurocognitive function of prefrontal cortex in ornithine transcarbamylase deficiency	Anderson, A	2020	CBSI
Behavioral and brain synchronization differences between expert and novice teachers when collaborating with students	Sun, B	2020	HbO2
Linguistic and non-linguistic non-adjacent dependency learning in early development	Van Der Kant, A	2020	HbO2 and HbR
The influence of maternal anxiety and depression symptoms on fNIRS brain responses to emotional faces in 5- and 7-month-old infants	Porto, J A	2020	HbO2
Cortical activity measured by functional near infrared spectroscopy during a theory of mind task in subjects with schizophrenia, bipolar disorder and healthy controls	Sayar-Akaslan, D	2020	HbO2
Evidence of fNIRS-Based Prefrontal Cortex Hypoactivity in Obesity and Binge-Eating Disorder	Rosch, S A	2020	HbO2
Neural Correlates of Age-Related Changes in Precise Grip Force Regulation: A Combined EEG-fNIRS Study	Berger, A	2020	HbO2
Development of the neural processing of vocal emotion during the first year of life	Zhao, C	2020	HbO2
Inner versus Overt Speech Production: Does This Make a Difference in the Developing Brain?	Stephan, F	2020	HbO2 and HbR
Facial and neural mechanisms during interactive disclosure of biographical information	Caniguera1, R	2020	HbO2 and HbR
The relationship of cortical activity induced by pain stimulation with clinical and cognitive features of somatic symptom disorder: A controlled fNIRS study	Colak, B	2020	HbO2
Functional Near-infrared Spectroscopy Reveals the Compensatory Potential of Pre-frontal Cortical Activity for Standing Balance in Young and Older Adults	St George,R J	2020	HbO2
People With Parkinson’s Disease Exhibit Reduced Cognitive and Motor Cortical Activity When Undertaking Complex Stepping Tasks Requiring Inhibitory Control	Pelicioni, P HS	2020	HbO2 and HbR
Similar activation patterns in the prefrontal cortex for Chinese and Japanese verbal fluency tests with syllable cues as revealed by near-infrared spectroscopy	Song, M	2020	HbO2
Neural correlates of mindful emotion regulation in high and low ruminators	Rosenbaum, D	2020	CBSI
Increased prefrontal oxygenation prior to and at the onset of over-ground locomotion in humans	Matsukawa, K	2020	HbO2
Hemodynamic and behavioral peculiarities in response to emotional stimuli in children with attention deficit hyperactivity disorder: An fNIRS study	Mauri, M	2020	HbO2 and HbR
Neural alignment during face-to-face spontaneous deception: Does gender make a difference?	Chen, M	2020	HbO2
Which obstacle attributes place additional demands on higher-level cognitive function in patients with Parkinson's disease?	Sharon, T	2020	CBSI

Dyad sex composition effect on inter-brain synchronization in face-to-face cooperation	Li, Y	2020	HbO2
Different strategies, distinguished cooperation efficiency, and brain synchronization for couples: An fNIRS-based hyperscanning study	Tang, Y	2020	HbO2
Exhaustion disorder and altered brain activity in frontal cortex detected with fNIRS	Skau, S	2020	HbO2
Interpersonal Neural Synchronization During Cooperative Behavior of Basketball Players: A fNIRS-Based Hyperscanning Study	Li, L	2020	HbO2
Interpersonal brain synchronization with instructor compensates for learner's sleep deprivation in interactive learning	Pan, Y	2020	CBSI
Positive Mood while Exercising Influences Beneficial Effects of Exercise with Music on Prefrontal Executive Function: A fNIRS Study	Suwabe, K	2020	HbO2
Cortical activity during social acceptance and rejection task in social anxiety disorder: A controlled fNIRS study	Kir, Y	2020	HbO2
Using Functional Near-Infrared Spectroscopy to Assess Brain Activation Evoked by Guilt and Shame	Duan, L	2020	HbO2
Human Discrimination and Categorization of Emotions in Voices: A Functional Near-Infrared Spectroscopy (fNIRS) Study	Gruber, T	2020	HbO2 and HbR
Auditory and Visual Response Inhibition in Children with Bilateral Hearing Aids and Children with ADHD	Bell, L	2020	HbO2 and HbR
Self–other overlap and interpersonal neural synchronization serially mediate the effect of behavioral synchronization on prosociality	Feng, X	2020	HbO2
Functional near-infrared spectroscopy (fNIRS) as a tool to assist the diagnosis of major psychiatric disorders in a Chinese population	Wei, Y	2020	HbO2 and HbR
Social exclusion influences conditioned fear acquisition and generalization: A mediating effect from the medial prefrontal cortex	Dou, H	2020	HbO2
Uncovering electrophysiological and vascular signatures of implicit emotional prosody	Steber, S	2020	HbO2 and HbR
The "gift effect" on functional brain connectivity. Inter-brain synchronization when prosocial behavior is in action	Balconi, M	2020	HbO2 and HbR
The Brain Differentially Prepares Inner and Overt Speech Production: Electrophysiological and Vascular Evidence	Stephan, F	2020	HbO2 and HbR
Developmental Differences in Cortical Activation During Action Observation, Action Execution and Interpersonal Synchrony: An fNIRS Study	Su, WC	2020	HbO2
The averaged inter-brain coherence between the audience and a violinist predicts the popularity of violin performanc	Hou, Y	2020	HbO2
Instructor-learner brain coupling discriminates between instructional approaches and predicts learning	Pan, Y	2020	CBSI
Functional lateralization of arithmetic processing in the intraparietal sulcus is associated with handedness	Artemenko, C	2020	CBSI
Affiliative bonding between teachers and students through interpersonal synchronisation in brain activity	Zheng, L	2020	HbO2
Effects of Tai Chi Chuan on Inhibitory Control in Elderly Women: An fNIRS Study	Yang, Y	2020	HbO2
How the Brain Understands Spoken and Sung Sentences	Rossi, S	2020	HbO2 and HbR
Stimulus modality influences the acquisition and use of the rule-based strategy and the similarity-based strategy in category learning	Wu, J	2020	Not Stated
Fixed and flexible: Dynamic prefrontal activations and working memory capacity relationships vary with memory demand	Shah, A M	2020	HbO2
Autism Symptoms Modulate Interpersonal Neural Synchronization in Children with Autism Spectrum Disorder in Cooperative Interactions	Wang, Q	2020	HbO2

Table S4 List of papers reviewed to determine signal usage.

Channel Configurations

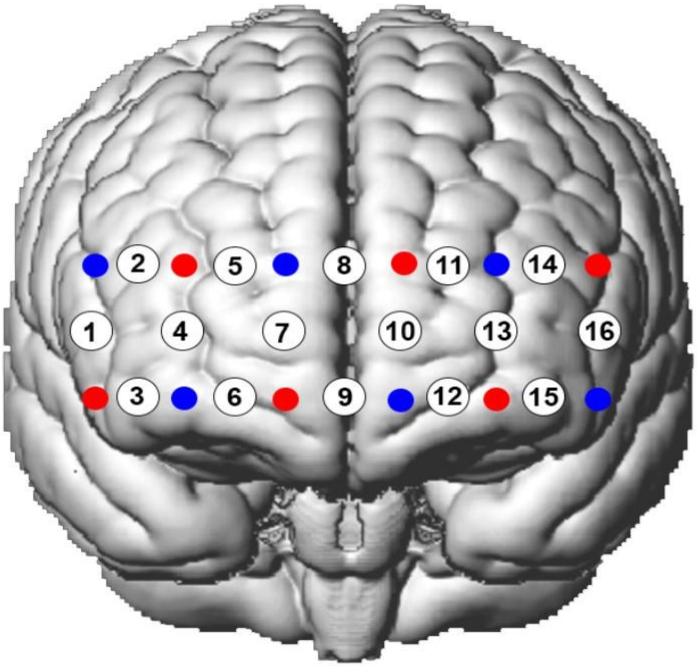

Figure S11 Hitachi Channel Configuration. Used to acquire resting state fNIRS data for the formation of synthetic data.

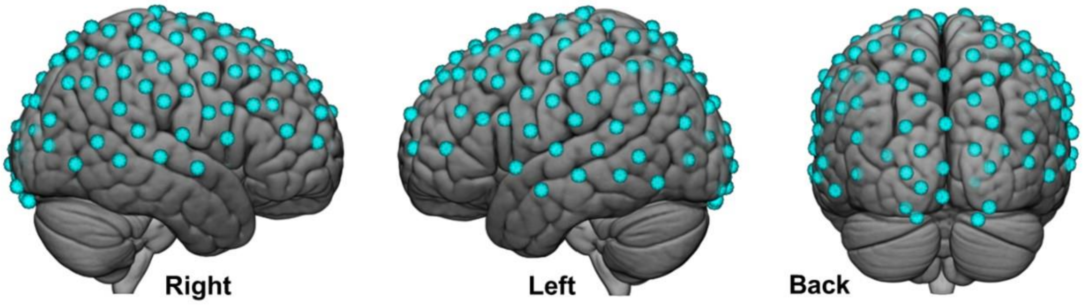

Figure S12 Shimadzu LABNIRS Channel Configuration. Used to acquire data from finger tapping experiment.

MNI Channel Locations

Channel	MNI	Anatomic Location	Overlap Percentage
1	-35 63 4	Frontal_Sup_L	0.36331
1		Frontal_Sup_Orb_L	0.0071942
1		Frontal_Mid_L	0.45324
1		Frontal_Mid_Orb_L	0.17626
2	40 61 7	Frontal_Sup_R	0.17803
2		Frontal_Mid_R	0.74242
2		Frontal_Mid_Orb_R	0.079545

3	-24 65 19	Frontal_Sup_L	0.89051
3		Frontal_Mid_L	0.10949
4	28 64 21	Frontal_Sup_R	0.62016
4		Frontal_Mid_R	0.37984
5	-12 62 34	Frontal_Sup_L	0.57875
5		Frontal_Sup_Medial_L	0.42125
6	15 61 34	Frontal_Sup_R	0.52874
6		Frontal_Mid_R	0.065134
6		Frontal_Sup_Medial_R	0.40613
7	-23 52 10	Frontal_Sup_L	0.7713
7		Frontal_Mid_L	0.2287
8	2 53 44	Frontal_Sup_Medial_L	0.56723
8		Frontal_Sup_Medial_R	0.43277
9	25 51 42	Frontal_Sup_R	0.76682
9		Frontal_Mid_R	0.23318
10	-49 38 26	Frontal_Mid_L	0.53383
10		Frontal_Inf_Tri_L	0.46617
11	-33 41 43	Frontal_Sup_L	0.25225
11		Frontal_Mid_L	0.74775
12	-12 45 52	Frontal_Sup_L	0.58704
12		Frontal_Sup_Medial_L	0.41296
13	14 44 53	Frontal_Sup_R	0.54688
13		Frontal_Sup_Medial_R	0.45313
14	34 41 45	Frontal_Sup_R	0.13122
14		Frontal_Mid_R	0.86878
15	50 38 30	Frontal_Mid_R	0.688
15		Frontal_Inf_Tri_R	0.312
16	-55 26 25	Frontal_Mid_L	0.017123
16		Frontal_Inf_Oper_L	0.058219
16		Frontal_Inf_Tri_L	0.92466

17	-43 29 45	Frontal_Mid_L	1
18	-22 33 57	Frontal_Sup_L	0.58065
18		Frontal_Mid_L	0.41935
19	1 34 58	Supp_Motor_Area_L	0.032847
19		Frontal_Sup_Medial_L	0.54015
19		Frontal_Sup_Medial_R	0.42701
20	24 32 58	Frontal_Sup_R	0.92607
20		Frontal_Mid_R	0.038911
20		Frontal_Sup_Medial_R	0.035019
21	44 29 47	Frontal_Mid_R	1
22	56 27 29	Frontal_Mid_R	0.13889
22		Frontal_Inf_Oper_R	0.24653
22		Frontal_Inf_Tri_R	0.61458
23	-61 10 2	Frontal_Inf_Oper_L	0.31399
23		Frontal_Inf_Tri_L	0.05802
23		Rolandic_Oper_L	0.22867
23		Temporal_Sup_L	0.157
23		Temporal_Pole_Sup_L	0.24232
24	-59 15 25	Precentral_L	0.21502
24		Frontal_Inf_Oper_L	0.43003
24		Frontal_Inf_Tri_L	0.35495
25	-49 18 45	Precentral_L	0.22433
25		Frontal_Mid_L	0.77186
25		Frontal_Inf_Oper_L	0.0038023
26	-34 21 59	Frontal_Mid_L	1
27	-13 24 66	Frontal_Sup_L	0.57244
27		Supp_Motor_Area_L	0.35336
27		Frontal_Sup_Medial_L	0.074205
28	14 24 66	Frontal_Sup_R	0.3007
28		Supp_Motor_Area_R	0.51049
28		Frontal_Sup_Medial_R	0.18881

29	34 21 60	Frontal_Sup_R	0.34615
29		Frontal_Mid_R	0.65385
30	50 18 47	Precentral_R	0.13127
30		Frontal_Mid_R	0.79537
30		Frontal_Inf_Oper_R	0.073359
31	61 15 29	Precentral_R	0.35484
31		Frontal_Inf_Oper_R	0.49462
31		Frontal_Inf_Tri_R	0.15054
32	64 12 8	Precentral_R	0.056478
32		Frontal_Inf_Oper_R	0.3588
32		Frontal_Inf_Tri_R	0.0299
32		Rolandic_Oper_R	0.39867
32		Temporal_Sup_R	0.046512
32		Temporal_Pole_Sup_R	0.10963
33	-66 -5 -8	Temporal_Sup_L	0.30033
33		Temporal_Mid_L	0.69967
34	-65 1 20	Precentral_L	0.14194
34		Frontal_Inf_Oper_L	0.1129
34		Rolandic_Oper_L	0.087097
34		Postcentral_L	0.65806
35	-58 3 40	Precentral_L	0.70803
35		Postcentral_L	0.29197
36	-43 9 57	Precentral_L	0.35317
36		Frontal_Mid_L	0.64683
37	-23 12 68	Frontal_Sup_L	0.73448
37		Frontal_Mid_L	0.26552
38	0 12 69	Supp_Motor_Area_L	0.58966
38		Supp_Motor_Area_R	0.41034
39	23 12 69	Frontal_Sup_R	0.89619
39		Supp_Motor_Area_R	0.10381
40	43 9 59	Frontal_Mid_R	1
41	59 4 44	Precentral_R	0.88803

41		Postcentral_R	0.11197
42	67 2 25	Precentral_R	0.38835
42		Rolandic_Oper_R	0.0032362
42		Postcentral_R	0.60841
43	68 -4 -1	Rolandic_Oper_R	0.013158
43		Heschl_R	0.016447
43		Temporal_Sup_R	0.91447
43		Temporal_Pole_Sup_R	0.042763
43		Temporal_Mid_R	0.013158
44	-68 -12 12	Rolandic_Oper_L	0.056426
44		Postcentral_L	0.42947
44		SupraMarginal_L	0.018809
44		Heschl_L	0.025078
44		Temporal_Sup_L	0.47022
45	-64 -8 -35	Precentral_L	0.02381
45		Postcentral_L	0.95918
45		SupraMarginal_L	0.017007
46	-52 -5 54	Precentral_L	0.52191
46		Postcentral_L	0.47809
47	-34 -1 67	Precentral_L	0.47887
47		Frontal_Sup_L	0.3662
47		Frontal_Mid_L	0.15493
48	-14 1 74	Frontal_Sup_L	0.65677
48		Supp_Motor_Area_L	0.34323
49	13 1 74	Frontal_Sup_R	0.50323
49		Supp_Motor_Area_R	0.49677
50	33 -2 68	Precentral_R	0.092857
50		Frontal_Sup_R	0.73571
50		Frontal_Mid_R	0.17143
51	52 -5 57	Precentral_R	0.46639
51		Frontal_Mid_R	0.47899
51		Postcentral_R	0.054622
52	65 -8 39	Precentral_R	0.10727

52		Postcentral_R	0.85121
52		SupraMarginal_R	0.041522
53	70 -10 18	Rolandic_Oper_R	0.083591
53		Postcentral_R	0.56966
53		SupraMarginal_R	0.11146
53		Temporal_Sup_R	0.23529
54	-70 -25 0	Temporal_Sup_L	0.074074
54		Temporal_Mid_L	0.92593
55	-68 -21 27	Postcentral_L	0.3865
55		SupraMarginal_L	0.6135
56	-60 -19 48	Postcentral_L	0.46182
56		Parietal_Inf_L	0.21455
56		SupraMarginal_L	0.32364
57	-45 -15 64	Precentral_L	0.60569
57		Postcentral_L	0.39431
58	-24 -12 74	Precentral_L	0.6835
58		Frontal_Sup_L	0.3165
59	-1 -11 75	Supp_Motor_Area_L	0.3268
59		Supp_Motor_Area_R	0.35948
59		Paracentral_Lobule_L	0.29085
59		Paracentral_Lobule_R	0.022876
60	23 -12 75	Precentral_R	0.51792
60		Frontal_Sup_R	0.48208
61	44 -15 66	Precentral_R	0.84528
61		Frontal_Sup_R	0.0075472
61		Postcentral_R	0.14717
62	61 -19 51	Precentral_R	0.0037879
62		Postcentral_R	0.625
62		Parietal_Inf_R	0.018939
62		SupraMarginal_R	0.35227
63	69 -20 33	Postcentral_R	0.17241
63		SupraMarginal_R	0.82759

64	72 -24 6	Temporal_Sup_R	0.87963
64		Temporal_Mid_R	0.12037
65	-69 -35 16	SupraMarginal_L	0.19315
65		Temporal_Sup_L	0.60748
65		Temporal_Mid_L	0.19938
66	-65 -31 40	Parietal_Inf_L	0.084691
66		SupraMarginal_L	0.91531
67	-53 -28 58	Postcentral_L	0.76834
67		Parietal_Inf_L	0.23166
68	-35 -24 71	Precentral_L	0.60606
68		Postcentral_L	0.39394
69	-14 -23 78	Precentral_L	0.17757
69		Postcentral_L	0.17134
69		Paracentral_Lobule_L	0.65109
70	12 -23 79	Precentral_R	0.3511
70		Frontal_Sup_R	0.021944
70		Supp_Motor_Area_R	0.068966
70		Postcentral_R	0.10658
70		Paracentral_Lobule_R	0.45141
71	33 -25 73	Precentral_R	0.71242
71		Postcentral_R	0.28758
72	52 -28 60	Precentral_R	0.0038023
72		Postcentral_R	0.6692
72		Parietal_Sup_R	0.11027
72		Parietal_Inf_R	0.20913
72		SupraMarginal_R	0.0076046
73	65 -31 45	Parietal_Inf_R	0.087542
73		SupraMarginal_R	0.91246
74	70 -34 23	SupraMarginal_R	0.47531
74		Temporal_Sup_R	0.52469
75	-68 -47 2	Temporal_Sup_L	0.025078
75		Temporal_Mid_L	0.97492

76	-66 -44 28	SupraMarginal_L	0.76115
76		Temporal_Sup_L	0.23885
77	-60 -42 48	Parietal_Inf_L	0.83333
77		SupraMarginal_L	0.16667
78	-44 -38 65	Postcentral_L	0.80934
78		Parietal_Sup_L	0.031128
78		Parietal_Inf_L	0.15953
79	-23 -35 76	Postcentral_L	0.90365
79		Parietal_Sup_L	0.093023
79		Paracentral_Lobule_L	0.0033223
80	23 -36 76	Precentral_R	0.092715
80		Postcentral_R	0.86093
80		Parietal_Sup_R	0.046358
81	43 -37 66	Precentral_R	0.0038314
81		Postcentral_R	0.83142
81		Parietal_Sup_R	0.16475
82	60 -41 51	Parietal_Sup_R	0.0074074
82		Parietal_Inf_R	0.72222
82		SupraMarginal_R	0.27037
83	67 -43 33	Parietal_Inf_R	0.0064935
83		SupraMarginal_R	0.82468
83		Angular_R	0.12013
83		Temporal_Sup_R	0.048701
84	69 -47 7	Temporal_Sup_R	0.093851
84		Temporal_Mid_R	0.90615
85	-65 -56 14	SupraMarginal_L	0.053156
85		Angular_L	0.013289
85		Temporal_Sup_L	0.21262
85		Temporal_Mid_L	0.72093
86	-61 -54 37	Parietal_Inf_L	0.26224
86		SupraMarginal_L	0.38112
86		Angular_L	0.35664
87	-51 -51 55	Parietal_Inf_L	1

88	-33 -48 69	Postcentral_L	0.15468
88		Parietal_Sup_L	0.84532
89	32 -48 70	Postcentral_R	0.4296
89		Parietal_Sup_R	0.5704
90	50 -51 56	Parietal_Sup_R	0.20553
90		Parietal_Inf_R	0.74308
90		Angular_R	0.051383
91	61 -53 40	Parietal_Inf_R	0.45357
91		SupraMarginal_R	0.16071
91		Angular_R	0.38571
92	65 -56 18	Occipital_Mid_R	0.0033557
92		Angular_R	0.12752
92		Temporal_Sup_R	0.2953
92		Temporal_Mid_R	0.57383
93	-59 -68 3	Occipital_Mid_L	0.1162
93		Occipital_Inf_L	0.080986
93		Temporal_Mid_L	0.74296
93		Temporal_Inf_L	0.059859
94	-55 -62 41	Parietal_Inf_L	0.23792
94		Angular_L	0.76208
95	-43 -59 58	Parietal_Sup_L	0.24481
95		Parietal_Inf_L	0.60166
95		Angular_L	0.15353
96	-24 -57 71	Parietal_Sup_L	0.96886
96		Precuneus_L	0.031142
97	24 -57 71	Parietal_Sup_R	1
98	41 -59 59	Parietal_Sup_R	0.48908
98		Parietal_Inf_R	0.34061
98		Angular_R	0.17031
99	54 -62 43	Parietal_Inf_R	0.3253
99		Angular_R	0.6747

100	58 -69 5	Occipital_Mid_R	0.1032
100		Temporal_Mid_R	0.80783
100		Temporal_Inf_R	0.088968
101	-47 70 43	Occipital_Mid_L	0.14602
101		Parietal_Inf_L	0.0088496
101		Angular_L	0.84513
102	-33 -68 59	Parietal_Sup_L	0.82456
102		Parietal_Inf_L	0.052632
102		Angular_L	0.12281
103	-14 -66 67	Parietal_Sup_L	0.45255
103		Precuneus_L	0.54745
104	14 -66 67	Parietal_Sup_R	0.72901
104		Precuneus_R	0.27099
105	32 -67 60	Parietal_Sup_R	0.84286
105		Parietal_Inf_R	0.07619
105		Angular_R	0.080952
106	46 -70 45	Angular_R	1
107	-23 -75 57	Parietal_Sup_L	0.9207
107		Parietal_Inf_L	0.035242
107		Precuneus_L	0.044053
108	1 -73 60	Precuneus_L	0.48606
108		Precuneus_R	0.51394
109	24 -74 57	Parietal_Sup_R	1
110	-13 -82 50	Occipital_Sup_L	0.51341
110		Parietal_Sup_L	0.36782
110		Precuneus_L	0.11877
111	15 -82 50	Cuneus_R	0.34496
111		Occipital_Sup_R	0.054264
111		Parietal_Sup_R	0.5155
111		Precuneus_R	0.085271
112	-23 -89 38	Occipital_Sup_L	0.75636
112		Occipital_Mid_L	0.23273

112		Parietal_Inf_L	0.010909
113	1 -88 40	Cuneus_L	0.57813
113		Cuneus_R	0.23438
113		Occipital_Sup_L	0.10938
113		Occipital_Sup_R	0.0039063
113		Precuneus_L	0.050781
113		Precuneus_R	0.023438
114	24 -88 38	Cuneus_R	0.17228
114		Occipital_Sup_R	0.74532
114		Occipital_Mid_R	0.082397
115	-32 -91 24	Occipital_Sup_L	0.18462
115		Occipital_Mid_L	0.81538
116	-12 -96 29	Cuneus_L	0.20073
116		Occipital_Sup_L	0.79927
117	14 -95 29	Cuneus_L	0.053571
117		Cuneus_R	0.19286
117		Occipital_Sup_R	0.75357
118	32 -91 25	Occipital_Sup_R	0.41065
118		Occipital_Mid_R	0.58935
119	-22 -99 17	Occipital_Sup_L	0.4684
119		Occipital_Mid_L	0.5316
120	0 -99 20	Calcarine_L	0.086957
120		Cuneus_L	0.53986
120		Cuneus_R	0.028986
120		Occipital_Sup_L	0.27174
120		Occipital_Sup_R	0.072464
121	23 -98 18	Cuneus_R	0.044444
121		Occipital_Sup_R	0.87407
121		Occipital_Mid_R	0.081481
122	-13 -103 9	Occipital_Sup_L	0.41751
122		Occipital_Mid_L	0.58249
123	13 -102 10	Calcarine_L	0.17705
123		Calcarine_R	0.12131

123		Cuneus_L	0.02623
123		Cuneus_R	0.2623
123		Occipital_Sup_R	0.41311
124	-23 -101 -2	Calcarine_L	0.10566
124		Occipital_Mid_L	0.73962
124		Occipital_Inf_L	0.15472
125	-1 -103 2	Calcarine_L	0.77667
125		Calcarine_R	0.0033333
125		Cuneus_L	0.026667
125		Occipital_Sup_L	0.1
125		Occipital_Mid_L	0.093333
126	22 -101 -1	Calcarine_R	0.32281
126		Lingual_R	0.25263
126		Occipital_Sup_R	0.1193
126		Occipital_Mid_R	0.098246
126		Occipital_Inf_R	0.20702
127	-14 -102 -9	Calcarine_L	0.34737
127		Lingual_L	0.32982
127		Occipital_Mid_L	0.21754
127		Occipital_Inf_L	0.10526
128	12 -101 -8	Calcarine_L	0.25818
128		Calcarine_R	0.16364
128		Lingual_R	0.57818
129	-49 46 7	Frontal_Mid_L	0.23105
129		Frontal_Mid_Orb_L	0.039711
129		Frontal_Inf_Tri_L	0.72202
129		Frontal_Inf_Orb_L	0.0072202
130	52 45 12	Frontal_Mid_R	0.57348
130		Frontal_Inf_Tri_R	0.42652
131	-47 -83 12	Occipital_Mid_L	1
132	46 -83 13	Occipital_Mid_R	0.94215
132		Temporal_Mid_R	0.057851
133	-47 -80 28	Occipital_Mid_L	0.67355
133		Angular_L	0.30165

133		Temporal_Mid_L	0.024793
134	45 -79 29	Occipital_Mid_R	0.84279
134		Angular_R	0.1441
134		Temporal_Mid_R	0.0131

Table S5 MNI coordinates of channels and corresponding anatomical locations for Shimadzu Configuration.

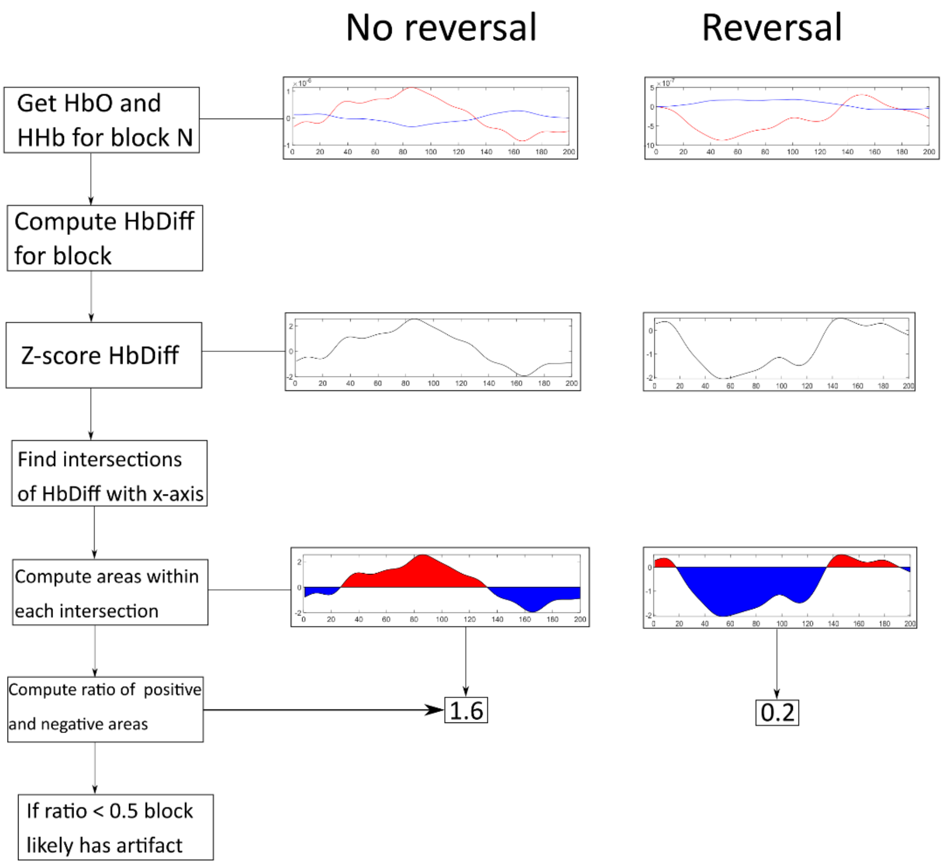

Figure S13 Method of detecting HbO_2 and HHb reversal pattern. Example output for typical and reversed data is shown. The red and blue shaded regions refer to the positive and negative areas of the Z-scored Hb_{Diff} signal.

N1 Synthetic data to test the reversal pattern identification method was generated by reversing the polarity of HbO_2 and HHb of the task-related HRF for three random task blocks in each dataset for the high SNR signal (see Fig. 2 in the main text). We then tested the method with a ratio threshold of 1, which included more non-reversed blocks. Lowering this to 0.5 was able to improve the specificity of the method.

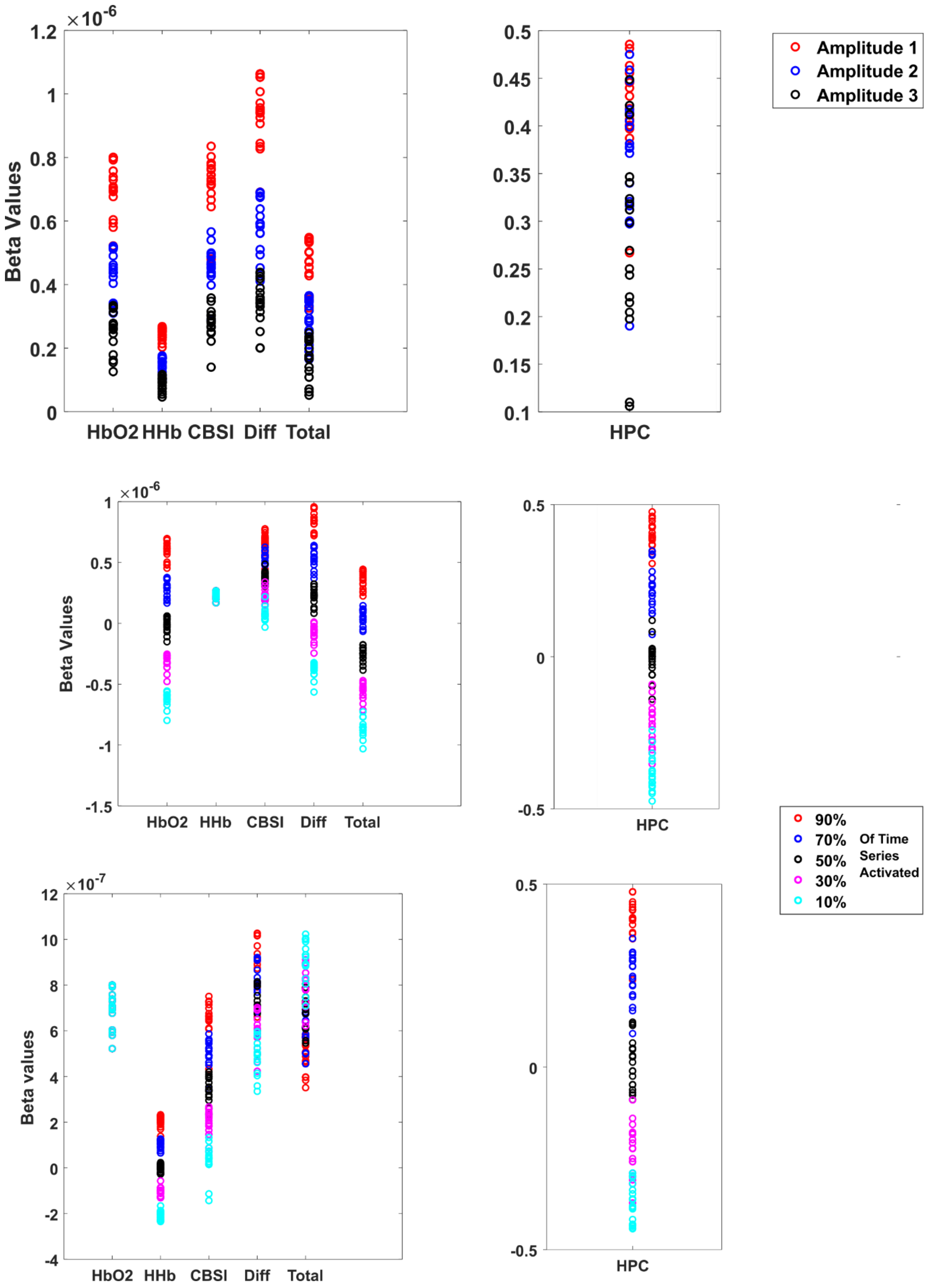

Figure S14 Raw beta values obtained from the synthetic analysis. The top panel is from the SNR test, the middle panel is the result from reversing the HHb signal, and the bottom is reversing the HbO_2 signal.

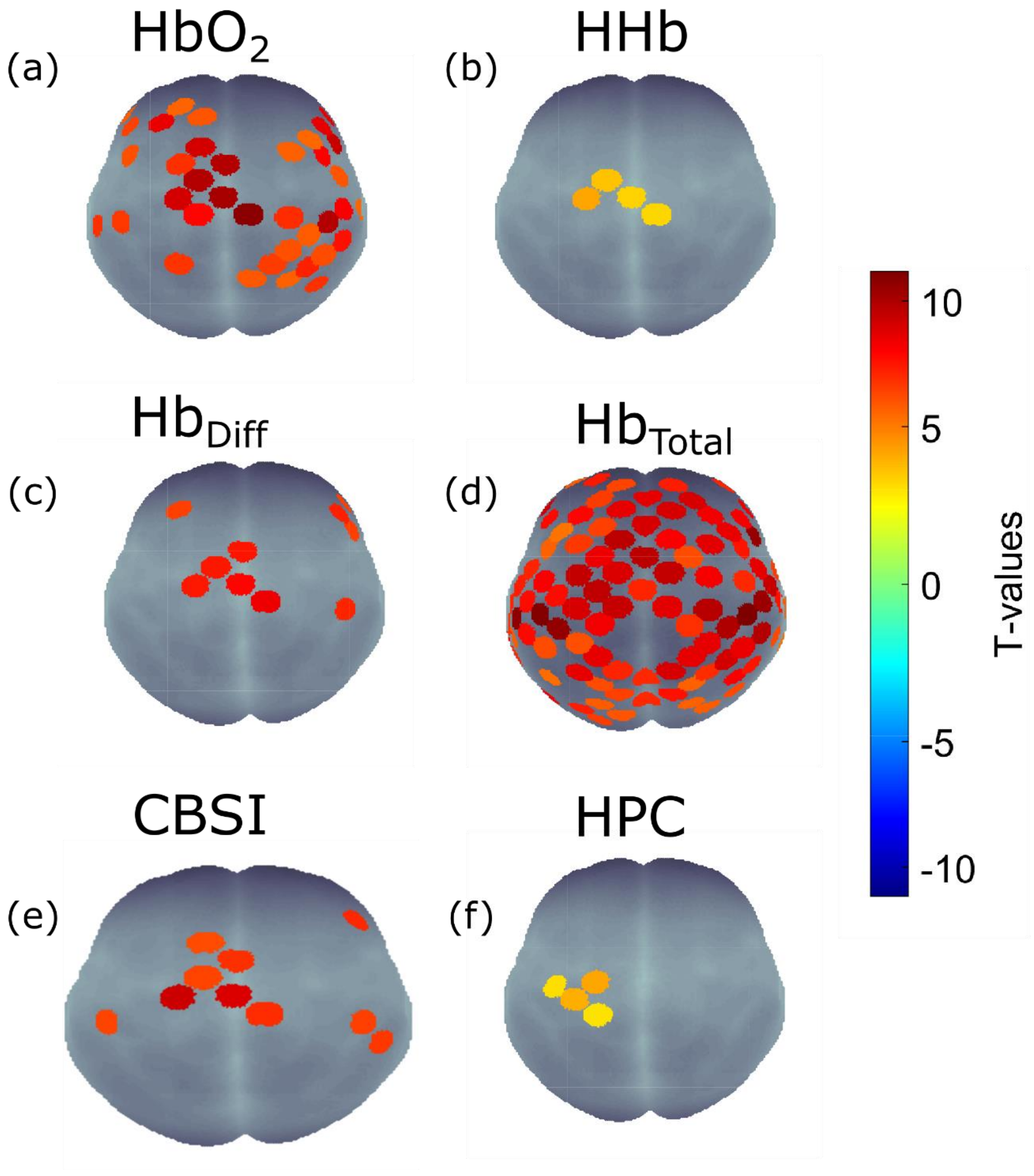

Figure S15. Effect of changing p-value on each signal. When reducing the p-value the number of channels also reduced. (A) HbO_2 ($p\leq 10^{-6}$), (B) HHb ($p\leq 0.05$), (C) Hb_{Diff} ($p\leq 10^{-6)}$, (D) Hb_{Tot} ($p\leq 10^{-6)}$, (E)CBSI ($p\leq 10^{-6)}$, (F)HPC ($p\leq 0.05$)

HHb Results for positive increase

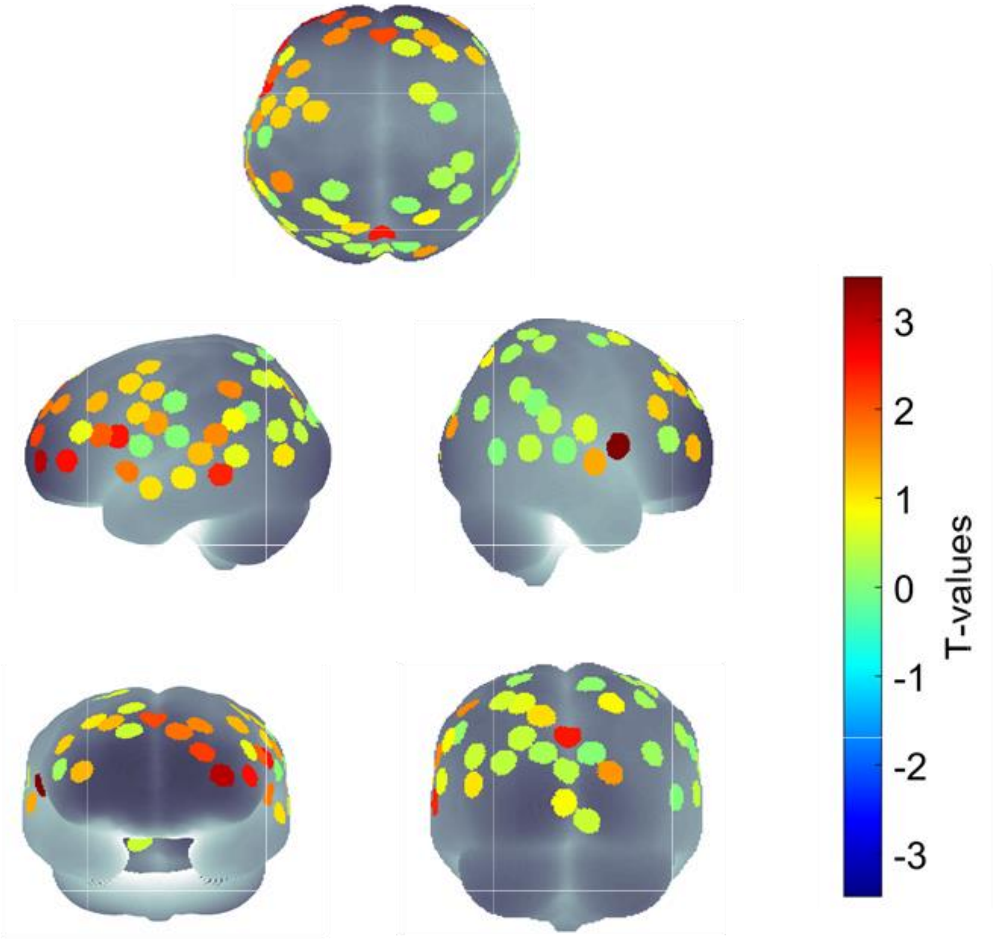

Figure S16 Results for HHb with the reverse contrast; finding where the HHb signal is increasing.
